# Antigestagens Mediate the Expression of Decidualization Markers, Extracellular Matrix Factors and Connexin 43 in Decidualized Dog Uterine Stromal (DUS) Cells

**DOI:** 10.3390/ani12070798

**Published:** 2022-03-22

**Authors:** Ali Kazemian, Miguel Tavares Pereira, Bernd Hoffmann, Mariusz P. Kowalewski

**Affiliations:** 1 Institute of Veterinary Anatomy, Vetsuisse Faculty, University of Zurich, 8057 Zurich, Switzerland; ali.kazemian@uzh.ch (A.K.); miguel.tavarespereira@uzh.ch (M.T.P.); 2 Clinic for Obstetrics, Gynaecology and Andrology of Large and Small Animals, Faculty of Veterinary Medicine, Justus Liebig University, 35392 Giessen, Germany; bernd.hoffmann@vetmed.uni-giessen.de; 3 Center for Clinical Studies (ZKS), Vetsuisse Faculty, University of Zurich, 8057 Zurich, Switzerland

**Keywords:** dog (*Canis lupus familiaris*), dog uterine stromal (DUS) cells, decidualization, nuclear progesterone receptor (PGR), antigestagens

## Abstract

**Simple Summary:**

Adequate embryo-maternal communication is essential for a successful pregnancy. In the dog, this interaction is intimately associated with maternal stroma-derived decidual cells, the only cell population in the canine placenta expressing the nuclear progesterone receptor (PGR) and, therefore, sensitive to the circulating progesterone levels. Prepartum decrease of progesterone or clinical application of PGR blockers (antigestagens, e.g., aglepristone and mifepristone) induce placental release of luteolytic factors and terminate pregnancy. However, the importance of progesterone for decidual cells functionality has not been fully elucidated. Therefore, we investigated the effects of PGR blockers on the expression of markers of decidualization and cellular viability, as well as on epithelial and mesenchymal factors in in vitro decidualized dog uterine stromal (DUS) cells. Decidualization increased the expression of the respective markers, including factors involved in cell growth and prostaglandin synthesis. Their expression was suppressed by the application of antigestagens. Additionally, the expression of factors involved in tissue remodeling and cell-cell communication was increased, and antiproliferative and proapoptotic effects were induced in decidualized cells. Altogether, progesterone signaling appears to be crucial for modulating decidual cells physiology and biological activity, and thus for the maintenance of pregnancy.

**Abstract:**

Feto-maternal communication in the dog involves the differentiation of stromal cells into decidual cells. As the only placental cells expressing the nuclear progesterone (P4) receptor (PGR), decidual cells play crucial roles in the maintenance and termination of pregnancy. Accordingly, to investigate possible PGR-mediated mechanisms in canine decidual cells, in vitro decidualized dog uterine stromal (DUS) cells were treated with functional PGR-blockers, mifepristone and aglepristone. Effects on decidualization markers, epithelial and mesenchymal factors, and markers of cellular viability were assessed. Decidualization increased the expression of *PTGES*, *PGR*, *IGF1*, and *PRLR,* along with *ECM1*, *COL4* and *CX43,* but downregulated *IGF2*. DUS cells retained their mesenchymal character, and the expression of COL4 indicated the mesenchymal-epithelial transformation. Antigestagen treatment decreased the availability of *PTGES*, *PRLR*, *IGF1* and PGR. Furthermore, antigestagens decreased the mRNA and protein expression of CX43, and transcriptional levels of *ECM1* and *COL4*. Additionally, antigestagens increased levels of activated-CASP3 (a proapoptotic factor), associated with lowered levels of PCNA (a proliferation marker). These data reveal important aspects of the functional involvement of PGR in canine decidual cells, regarding the expression of decidualization markers and acquisition of epithelial-like characteristics. Some of these mechanisms may be crucial for the maintenance and/or termination of canine pregnancy.

## 1. Introduction

The establishment of pregnancy depends on well-coordinated embryo-maternal crosstalk that supports the formation of the placenta. In several mammals, this crosstalk also includes the development of an anti-luteolytic signal involved in the maternal recognition of pregnancy. However, in the domestic dog (*Canis lupus familiaris*), which lacks luteolysis in non-pregnant animals, maternal recognition of pregnancy is associated with morphofunctional changes of the uterine compartment induced by the presence of free-floating embryos [1,2]. These changes are a pre-requisite for embryonic implantation and the subsequent formation of a shallow invasive endotheliochorial placenta [1,2].

Canine placentation is associated with the species-specific decidualization of maternal stromal cells [3]. Decidualization is also observed in other species, e.g., humans and rodents [4,5], and is characteristic of invasive placentation. While decidualization in humans is spontaneous in response to increasing circulating levels of P4 [6], embryo-driven stimuli are required for the induction of decidualization in the dog and in rodents [1,7,8]. Canine decidualization is associated with the modulated uterine expression of several factors referred to as decidualization markers, including insulin-like growth factor (IGF)-1 and -2, nuclear progesterone receptor (PGR), estrogen receptor alpha (ERα), prolactin receptor (PRLR), and members of the prostaglandin (PG) family, such as prostaglandin transporter (PGT), PGE2 synthase (PTGES), PGF2α synthase (PTGFS) and some of their receptors [1,2]. These changes in the stromal compartment culminate in the formation of highly specialized decidual cells, which are larger and more rounded than stromal cells, and are located around maternal blood vessels [3,9].

Decidual cells play key roles in embryo-maternal communication as the only cell population in the canine placenta expressing PGR, which is crucial for the maintenance of pregnancy, as well as ERα and oxytocin receptor (OXTR) [10,11,12]. Importantly, interfering with PGR function, e.g., by administration of PGR blockers (antigestagens), leads to an increased placental production of PGF2α and induction of luteolysis, unequivocally resulting in the interruption of pregnancy (pre-term parturition or abortion) [13,14,15,16].

By competing with P4 in binding to PGR, antigestagens interfere with P4 signaling [17]. Type II antigestagens, which are also used in veterinary practice, function as transdominant repressors, i.e., they activate PGR and induce its attachment to promoters of target genes, but inhibit the downstream cascades, e.g., by recruitment of corepressors to target gene promoters, thereby actively inhibiting their expression [18,19,20,21]. Consequently, type II antigestagens do not require the natural PGR ligand (P4) to exhibit their function [17]. This family of antigestagens is represented by mifepristone (RU483), and by its close derivative, aglepristone (RU534), which was developed for clinical use in veterinary medicine [18], and is currently the drug of choice for the interruption of unwanted pregnancies in bitches [22,23]. At the functional level, a recent transcriptomic study revealed several functional similarities between the canine term placenta and placentas from dogs in which abortion was induced with aglepristone at mid-pregnancy [24]. In both situations, termination of pregnancy was associated with increased apoptotic signaling, disruption of blood vessel function, modulation of immune response and increased lipid metabolism, whereas cell growth and adhesion were negatively affected [24]. However, the underlying molecular mechanisms with regard to the PGR-dependent embryo-maternal crosstalk need to be further elucidated.

A previously established immortalized cell line of dog uterine stromal (DUS) cells provides a reliable in vitro model to study the physiology of decidual cells [3]. These cells can be decidualized in vitro with cAMP, presenting an increased expression of decidualization markers, e.g., *IGF1, PTGES*, *PRLR* and *PGR* [3,9]. The in vitro decidualization of these cells also leads to upregulation of components of the extracellular matrix, e.g., ECM1, collagen (COL) 4, and connexin (CX) 43. While decidualized DUS cells retain the expression of mesenchymal markers like vimentin (VIM) [9,25], their upregulated expression of COL4, which marks the mesenchymal-epithelial transition of stromal cells, is of particular importance [25]. Also, this phenomenon is similar between the dog and other decidualization models [26,27]. Nevertheless, the possible contribution of P4/PGR signaling to the mesenchymal-epithelial transformation in decidualizing canine uterine stromal cells remains unclear.

Here, the hypothesis was tested that decidual cell-derived PGR, as an important element of embryo-maternal communication, plays regulatory roles in the underlying morpho-functional biological processes associated with canine-specific decidualization. In order to test this hypothesis, in vitro model of canine decidualization was employed to investigate the effects of mifepristone and aglepristone, two representatives of type II antigestagens, on decidualized DUS cells.

## 2. Materials and Methods

### 2.1. Cell Culture and In Vitro Experiments

The immortalized dog uterine stromal (DUS) cell line was used as the cell culture model in this study [3,25], following previously described procedures [3,9,25]. Briefly, cells were grown in 150 cm^2^ cell culture flasks (Corning, New York, NY, USA) in maintenance medium consisting of DMEM-High Glucose (4.5 g/L; Bio Concept, Allschwil, Switzerland; pH = 7.2–p7.4) with 10% heat inactivated fetal bovine serum (FBS; Thermo Fisher Scientific AG, Reinach, Switzerland), 100 U/mL penicillin and 100 μg/mL streptomycin (PAN Biotech, Aidenbach, Germany), and 1% insulin-transferrin-selenium (ITS; Corning from Thermo Fisher Scientific AG, Reinach, Switzerland), under standard culture conditions (i.e., 37 °C, 5% CO_2_ in air, in a humidified incubator). When 80 to 90% confluency was reached, cells were trypsinized and seeded into 6-well plates (TPP Techno Plastic Products AG, Trasandingen, Switzerland) at a concentration of 2 × 10^5^ cells per well, and were allowed to attach and grow for 24 h. Subsequently, cAMP-mediated decidualization was induced following the previously described protocol, using N6,2′-O-dibutyryladenosine-3′,5′-cyclicmonophosphate (“cAMP”) [3,9]. For this, cells were washed with pre-warmed PBS and incubated for 72 h with serum-free stimulation medium, i.e., maintenance medium in which FBS was replaced with 0.01% bovine serum albumin (BSA; SUB001, Canvax Biotech, Córdoba, Spain), containing 0.5 mM cAMP (D0627, Sigma-Aldrich Chemie GmbH, Buchs, Switzerland). Subsequently, decidualized cells were treated with type II antigestagens by culture in stimulation medium containing different concentrations (0.5 μM, 1 μM and 2.0 μM) of mifepristone (Sigma-Aldrich Chemie GmbH, Buchs, Switzerland), or aglepristone (Batch No: 2064665, kindly provided by Virbac, Tierarzneimittel GmbH, 23843 Bad Oldesloe, Germany). DUS cells incubated for the same length of time in stimulation medium without cAMP and PGR-blockers served as controls. All experiments were performed at least three times with cells derived from different passages for each experimental setup (biological replicates [28]).

### 2.2. RNA Isolation, Reverse Transcription (RT) and Semi-Quantitative (TaqMan) PCR

Experiments were stopped by washing with ice cold PBS and cells were harvested with TRIzol reagent (Invitrogen, Carlsbad, CA, USA), further used for total RNA isolation. Detailed protocols for subsequent preparations and semi-quantitative TaqMan PCR have been previously described [29,30]. In short, RNA concentration and purity were measured with a NanoDrop 2000 Spectrophotometer (Thermo Fisher Scientific AG, Reinach, Switzerland). For each sample, a total of 1.3 μg RNA was subjected to DNase treatment using RQ1 RNase-free DNase (Promega, Duebendorf, Switzerland) for removal of possible contamination with genomic DNA, and complementary DNA (cDNA) was synthesized using MultiScribe Reverse Transcriptase, with random hexamers applied as primers, along with other RT reagents (Applied Biosystems by Thermo Fisher, Waltham, MA, USA). For the semi-quantitation of selected gene expression, PCRs were run in duplicate using TaqMan Universal Master Mix in an automated ABI PRISM 7500 Sequence Detection System fluorometer (Applied Biosystems, by Thermo Fisher, Waltham, MA, USA). Autoclaved water or non-reverse transcribed RNA (RT-minus control) were used instead of cDNA as negative controls. The detailed description of primers and TaqMan probes used is listed in Table 1. Commercially available TaqMan systems were ordered from Applied Biosystems. When not available, primers and 6-carboxyfluorescein (6-FAM) and 6-carboxytetramethylrhodamine (TAMRA) labelled probes, constructed based on published coding sequences, were self-designed and purchased from Microsynth (Balgach, Switzerland). The efficiency of probes was evaluated to ensure approximately 100%, as previously described [29,31]. Relative gene expression was quantified with the ΔΔCt method, using KDM4A, EIF4H and PTK2, reported to be stably expressed in the canine uterus [32], as reference genes.

### 2.3. Immunofluorescence (IF) Staining, and Evaluation of Data Using CellProfiler

For immunofluorescence staining, DUS cells were seeded into Nunc Lab-Tek II Chamber Slides (Thermo Fisher Scientific AG, Reinach, Switzerland) at a concentration of 6 × 10^4^ cells per well. All in vitro experimental procedures with DUS cells were performed as described above. At the end of the incubation periods, cells were fixed with formaldehyde, which was added to the incubation medium to a final concentration of 2%, for 10 min at 37 °C. Afterwards, cells were washed two times for 5 min with cold PBST (PBS + 0.25% Triton X). Following fixation, chambers were removed, and slides were subjected to immunofluorescence staining following the previously published protocol [33]. Briefly, nonspecific binding was blocked with 10% goat serum diluted in PBST, and slides were incubated with primary antibodies for 2 h at ambient temperature. After being washed with PBST, slides were then incubated with secondary antibody for 1 h at ambient temperature. The detailed information regarding all antibodies used for immunostaining is listed in Table 2. To visualize the nuclei, 4′,6-diamidino-2-phenylindole (DAPI; Sigma-Aldrich Chemie GmbH, Buchs, Switzerland) was added to the secondary antibody at a final concentration of 1:1000. Post-fixation was performed with 2% formaldehyde and slides were mounted using Glycergel (Dako North America, Inc., Carpinteria, CA, USA). Slides were then subjected to microscopy analysis using a Leica DMI6000B fluorescence microscope equipped with a Leica K5 camera (Leica Microsystems, CMS GmbH, Breisgau, Germany). Negative controls were created by staining slides without primary or secondary antibodies. The quantification of the intensity of positive signals was performed as previously described [25]. Pictures of six randomly selected areas were taken with 63× dry magnification lens. Image analysis was performed with CellProfiler 3.0.0 software [34], by measuring the full intensity of each colorimetric signal (color channel) according to the emitted wavelength. The intensity of signals was then normalized to the number of cells in each field, quantified by the number of nuclei present. In all analyzed pictures, the separation between cells was confirmed to avoid overlapping of nuclei. Furthermore, to ensure a higher reliability of measurements, at least 200 cells were counted in each experimental group for each factor, and at least three experiments from consecutive passages were performed.

### 2.4. Protein Preparation and Western Blot Analysis

Western blot analysis was carried out according to the previously published protocol [15]. Cell cultures were washed with ice cold PBS and collected after adding NET-2 lysis buffer (50 mM Tris-HCl, pH 7.4, 300 mM NaCl, 0.05% NP-40) containing 10 μL/mL protease inhibitor cocktail (Sigma-Aldrich Chemie GmbH, Buchs, Switzerland). Further nuclear lysis and homogenization was performed through sonication with a Vibra-Cell 75186 (Sonics & Materials, Inc., Newtown, CT, USA) at 75W two times for 10 s. The concentration of protein in each sample was measured by Bradford assay in a Smart Spec Plus spectrophotometer (Bio-Rad Laboratories, Munich, Germany) and was normalized with sample buffer (25 mM Tris-Cl, pH 6.8, 1% SDS, 5% β-mercaptoethanol, 10% glycerol, 0.01% bromophenol blue). A total of 20 μg protein per sample was subjected to electrophoresis in a 12% polyacrylamide gel (Bio-Rad Laboratories). Proteins were transferred onto methanol-activated polyvinylidene difluoride (PVDF) membranes (Bio-Rad Laboratories), and non-specific binding sites were blocked by 5% low-fat dry milk diluted in PBST for 1 h at ambient temperature. Afterwards, membranes were probed with primary antibodies overnight at 4 °C. After washing with PBST, membranes were incubated with horseradish peroxidase (HRP)-conjugated secondary antibodies for 1 h at ambient temperature. The detailed information regarding all antibodies used for the western blot analysis can be found in Table 2. Signals were detected with the SuperSignal West Chemiluminescent Kit substrate (Thermo Fisher Scientific AG, Reinach, Switzerland) and visualized with a ChemiDoc XRS+ System and Image Lab Software (Bio-Rad Laboratories). Semi-quantification was performed with the ImageJ Software (US National Institutes of Health, Bethesda, Maryland, USA), using the standardized optical density of the target protein normalized against ACTB stained on re-blotted membranes.

### 2.5. Statistical Analysis

GraphPad 3.06 Software (GraphPad Software, San Diego, CA, USA) was used for all statistical analyses. Significant differences in time-course experiments between both experimental conditions (i.e., control vs. cAMP treatment) were evaluated with an unpaired two-tailed Student’s *t*-test. Further statistical analysis was performed with a parametric one-way ANOVA. When *p* was less than 0.05, ANOVA was followed by the Tukey-Kramer multiple comparisons post-hoc test. Numerical data is presented as mean +/− standard deviation.

## 3. Results

### 3.1. Time-Dependent Effects of Antigestagen Treatment on the In Vitro Expression of Decidualization Markers

**Figure 1:**
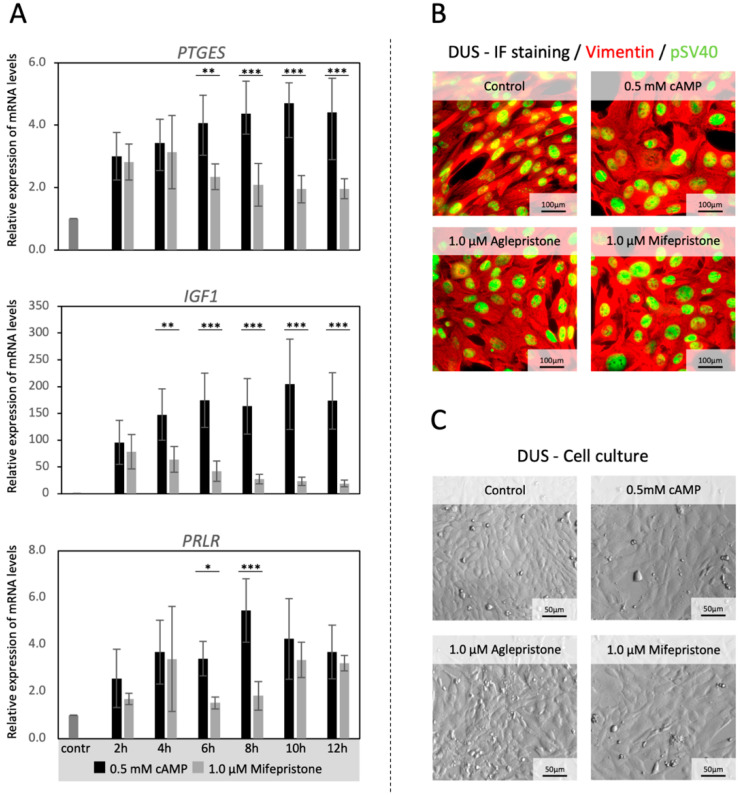
Time-dependent effects of mifepristone on the availability of decidualization markers in DUS cells. (**A**) Decidualized DUS cells (0.5 mM cAMP, 72 h) were treated with 1 μM mifepristone for up to 12 h. The relative gene expression of prostaglandin E2 synthase (*PTGES*), prolactin receptor (*PRLR*), and insulin-like growth factor (*IGF*)-1, was evaluated at different time points of treatment and compared with control cells. Two-tailed Student’s t-test was applied at each time point. Numerical data are presented as mean ± standard deviation (SD). Bars with different asterisks differ at: (*) *p* < 0.05, (**) *p* < 0.01, (***) *p* < 0.001. (**B**) Expression of vimentin (VIM, mesenchymal marker—red color) and pSV40 (marker of immortalization—green color) in DUS cells, observed by immunofluorescence (IF), after decidualization and/or treatment with antigestagens for 6 h. (**C**) Morphological appearance of DUS cells following in vitro decidualization and/or after 6 h treatment with 1 μM aglepristone or mifepristone.

**Figure 2:**
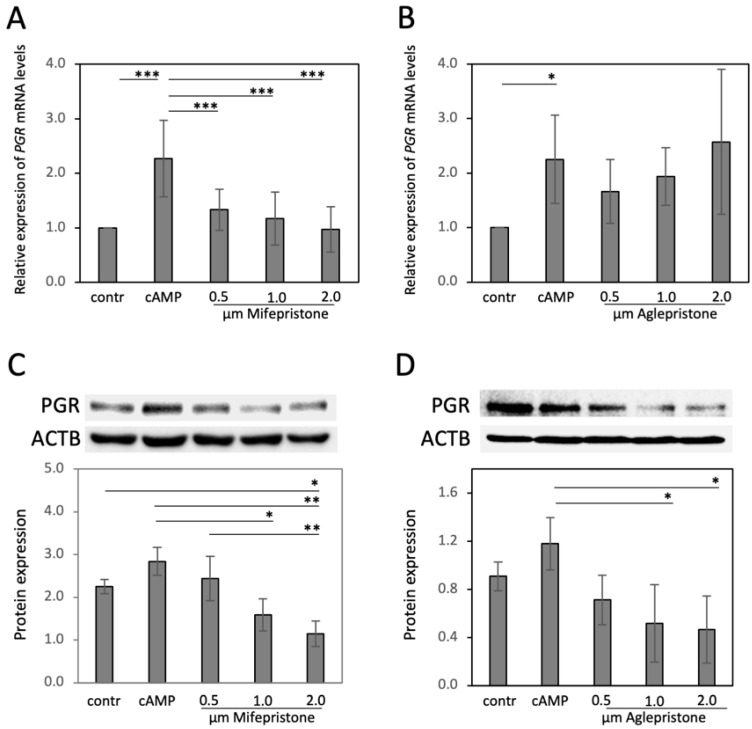
Antigestagen-mediated effects on the expression of PGR in decidualized DUS cells. DUS cells were decidualized with 0.5 mM cAMP for 72 h and consequently treated with increasing dosages of mifepristone or aglepristone for 6 h: (**A**,**B**) Relative expression of nuclear progesterone receptor (*PGR*) was determined by semi-quantitative real-time (TaqMan) PCR. (**C**,**D**) western blots were performed and optical densities of PGR (approx. 100 kDA) were normalized against ACTB (45 kDa). One-way ANOVA was applied, revealing: (**A**,**B**) *p* < 0.0001 and *p* = 0.0016 for mifepristone or aglepristone, respectively, and (**C**,**D**) *p* < 0.0001 and *p* = 0.01, respectively. This was followed by Tukey-Kramer multiple-comparisons test. Numerical data are presented as mean ± standard deviation (SD). Bars with different asterisks differ at: (*) *p* < 0.05, (**) *p* < 0.01, (***) *p* < 0.001.

The evaluation of time-dependent effects of an antigestagen on the transcriptional availability of major canine decidualization markers (*PTGES*, *IGF1* and *PRLR)* was performed by incubating decidualized DUS cells with 1 μΜ mifepristone for up to 12 h. This dosage was selected based on pilot experiments (included in Figure 1 and Figure 2). As expected, the expression of all target genes was significantly upregulated (*p* < 0.001) during in vitro decidualization (Figure 1A). The expression of *PTGES* was significantly decreased in treated cells compared with control samples from the 6 h time-point onwards (*p* < 0.01 for 6 h and *p* < 0.001 for all the other time points, Figure 1A). The *IGF1* was significantly lowered by 4 h (*p* < 0.01 Figure 1A), and remained suppressed (*p* < 0.001) until the 12 h observation time point. As for *PRLR*, although its expression varied greatly across the groups, it was significantly suppressed by mifepristone (*p* < 0.05) after 6 h and 8 h of treatment (*p* < 0.001, Figure 1A). Following these results, all further experiments with antigestagens were performed for 6 h, after the 72 h decidualization protocol. The morphological and biological properties of DUS cells were monitored throughout the experiments, and the cells were found to retain their mesenchymal character and vimentin expression, as well as the nuclear expression of the immortalization factor pSV40 [3] (Figure 1B). Their morphology changed during decidualization, becoming more rounded and larger in size, indicating the morphological mesenchymal-epithelial transition observed previously [3,25] (Figure 1C). No changes in morphology or confluence of cells were noted in response to antigestagens (Figure 1C).

### 3.2. Antigestagens Modulate the Expression of Decidualization Markers in DUS Cells

**Figure 3:**
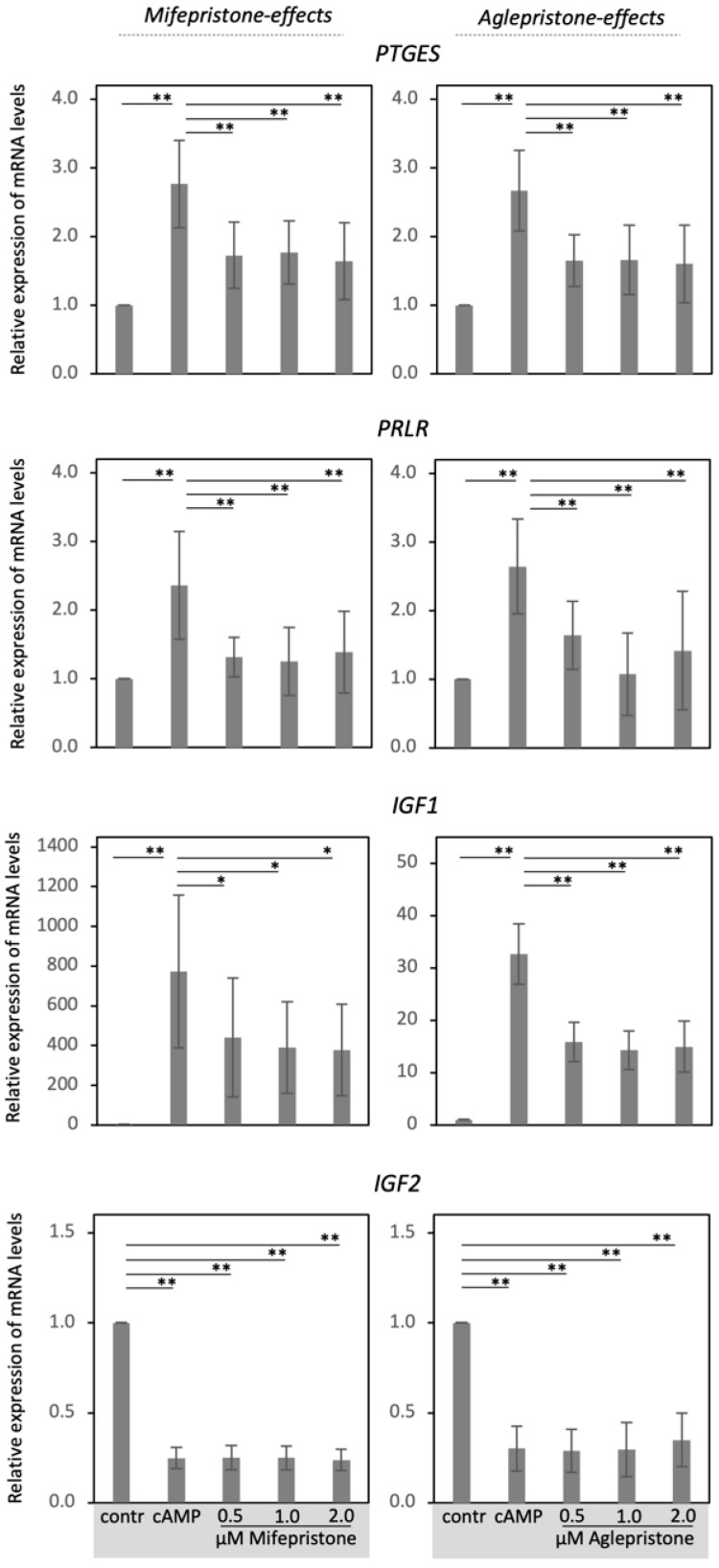
Antigestagen-mediated effects on the expression of decidualization markers in decidualized DUS cells. Relative expression of prostaglandin E2 synthase (*PTGES*), prolactin receptor (*PRLR*), insulin-like growth factor (*IGF*)-1, and -2, as determined by semi-quantitative real-time (TaqMan) PCR in DUS cells decidualized with 0.5 mM cAMP for 72 h and consequently treated with increasing dosages of mifepristone or aglepristone for 6 h. Effects of treatments were evaluated with a one-way ANOVA (*p* < 0.0001 for all the evaluated factors), followed by a Tukey-Kramer multiple-comparisons test. Numerical data are presented as mean ± standard deviation (SD). Bars with different asterisks differ at: (*) *p* < 0.01, (**) *p* < 0.001.

In the next experiments, decidualized DUS cells were treated with different dosages of mifepristone and aglepristone for 6 h, and the effects on selected decidualization markers (*PGR*, *PTGES*, *PRLR*, *IGF1*, *IGF2*) were assessed (Figure 2 and Figure 3). Decidualization increased the expression of *PGR* (*p* < 0.05, Figure 2A,B), *PTGES, PRLRL* and *IGF1* (all *p* < 0.001, Figure 3), but decreased *IGF2* availability (*p* < 0.001, Figure 3). Both mifepristone and aglepristone, at all applied concentrations, strongly suppressed detectable levels of *PTGES*, *PRLR* and *IGF1* (*p* < 0.01, statistical details in Figure 3). Interestingly, although mifepristone strongly suppressed the gene expression of *PGR* (*p* < 0.001, Figure 2A), aglepristone appeared less effective, showing considerable variation, with no significant effects upon the *PGR* mRNA abundance (*p* > 0.05, Figure 2B). However, in particular with higher dosages, mifepristone and aglepristone both significantly suppressed detectable levels of the PGR protein (*p* < 0.01, respectively, Figure 2C,D). The expression of *IGF2* did not change following mifepristone and aglepristone treatment, when compared with decidualized cells (*p* > 0.05, Figure 3). All statistical details are presented in Figure 2 (the whole blot figure is presented in the Supplementary File S1) and Figure 3.

### 3.3. Antigestagens Modulate the Expression of ECM Factors and CX43, but Not of Mesenchymal Markers

**Figure 4:**
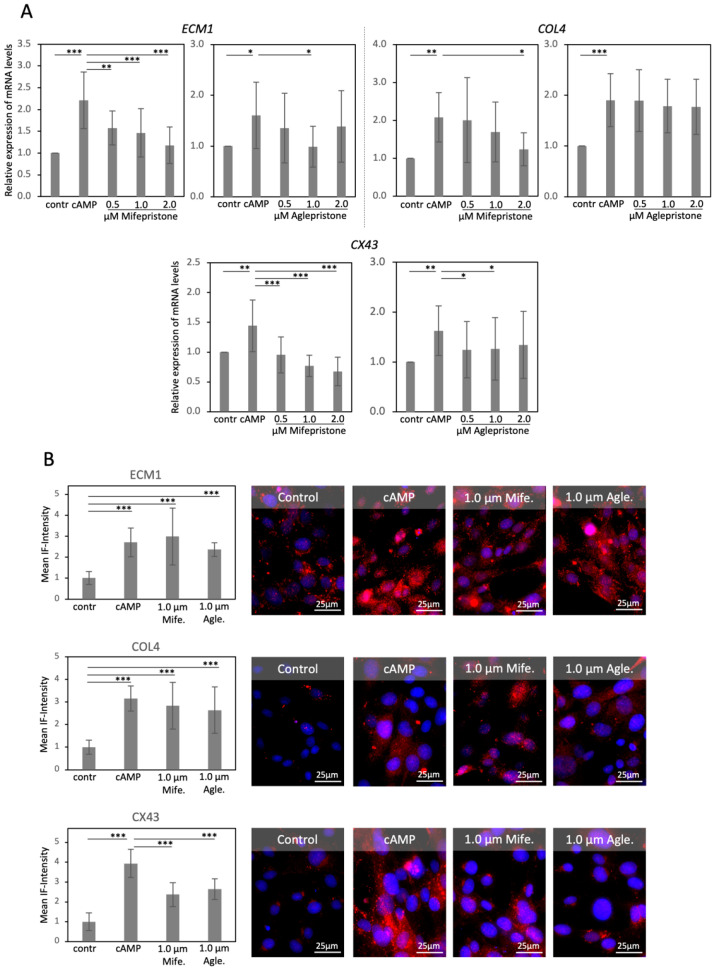
Antigestagen-mediated effects on the expression of extracellular matrix protein (ECM) 1, collagen (COL) 4, and connexin (CX) 43 in DUS cells. (**A**) Relative gene expression of *ECM1*, *COL4*, and *CX43*, as determined by semi-quantitative real-time (TaqMan) PCR in DUS cells decidualized with 0.5 mM cAMP for 72 h and consequently treated with increasing dosages of mifepristone or aglepristone for 6 h. (**B**) Quantification of protein expression of the selected factors as determined by immunofluorescence (IF) staining and corresponding representative figures. Effects of treatments in the transcriptional and protein availability of investigated factors were evaluated with a parametric one-way ANOVA, revealing: (**A**) *p* < 0.0001 and *p* = 0.0126 for *ECM1* in response to mifepristone or aglepristone, respectively; *p* = 0.0009 and *p* < 0.0001 for *COL4*, respectively; *p* < 0.0001 and *p* = 0.004 for *CX43*, respectively; in (**B**) *p* < 0.0001 for the mean IF-intensity of all the evaluated markers. The ANOVA tests were followed by a Tukey-Kramer multiple-comparisons test. Numerical data are presented as mean ± standard deviation (SD). Bars with different asterisks differ at: (*) *p* < 0.05, (**) *p* < 0.01, (***) *p* < 0.001. Mife. = Mifepristone, Agle. = Aglepristone.

Decidualization of DUS cells was associated with an increased abundance of transcripts for *ECM1* (*p* < 0.05), *COL4* (*p* < 0.01) and *CX43* (*p* < 0.01, Figure 4A). Treatment with mifepristone decreased the transcript abundance of *ECM1* and *CX43* at all tested concentrations (*p* < 0.01), while *COL4* showed lowered mRNA levels in response to the highest dosage of mifepristone (2 μM, *p* < 0.05, Figure 4A). With aglepristone, the expression of *ECM1* varied greatly following the treatment and proved to be significantly suppressed in response to 1 μM of the compound (*p* < 0.05, Figure 4A). Similarly, the expression of the mRNA encoding for *CX43* was suppressed in groups treated with 0.5 μM and 1 μM aglepristone (*p* < 0.05, Figure 4A), however, it did not modulate the levels of *COL4* mRNA (*p* > 0.05, Figure 4A).

At the protein level, decidualization led to the increased protein availability of ECM1, COL4 and CX43 (*p* < 0.001, Figure 4B). However, ECM1 and COL4 remained unaffected by the 6 h treatment with both antigestagens (*p* > 0.05, Figure 4B). This was different for CX43, the expression of which was significantly diminished in response to both antigestagens (*p* < 0.001, Figure 4B), mirroring the effects observed at the mRNA level.

**Figure 5:**
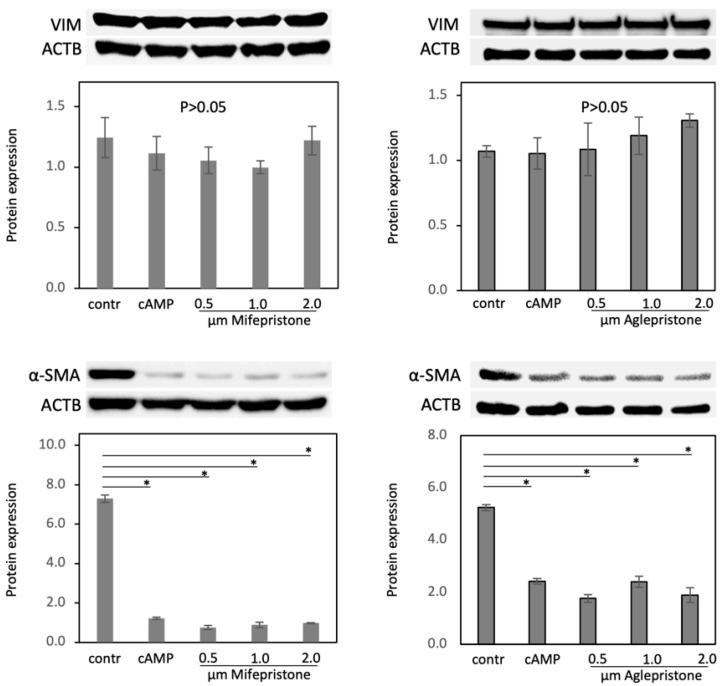
Expression of mesenchymal markers vimentin (VIM) and α-smooth muscle actin (α-SMA) in decidualized and antigestagen-treated DUS cells. Optical densities of VIM (54 kDa) and α-SMA (42 kDa) were normalized against ACTB (45 kDa). One-way ANOVA was applied, revealing: *p* > 0.05 for VIM in response to both mifepristone and aglepristone, and *p* < 0.0001 for α-SMA in the presence of mifepristone or aglepristone. The ANOVA tests were followed by Tukey-Kramer multiple-comparisons test. Numerical data are presented as mean ± standard deviation (SD). Bars with different asterisks differ at: (*) *p* < 0.001.

Finally, the protein expression of the mesenchymal markers VIM and α-SMA was investigated, and the effects of mifepristone and aglepristone were assessed in decidualized DUS cells (Figure 5). The expression of VIM was unaffected by decidualization, and remained stably expressed in antigestagens’ treated cells (*p* > 0.05, Figure 5 (the whole blot figure is presented in the Supplementary File S1)). As for α-SMA, despite being clearly detectable, it decreased during the decidualization process (*p* < 0.001, Figure 5) and remained unaffected in cells treated with both antigestagens.

### 3.4. Antigestagens Modulate the Expression of PCNA and a-CASP3 in Decidualized DUS Cells

**Figure 6:**
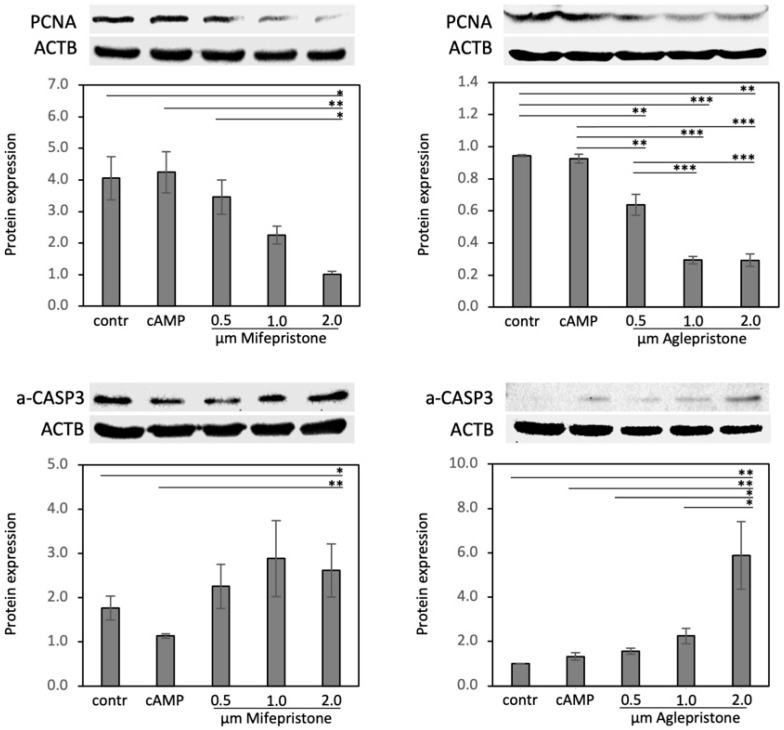
Antigestagen-mediated effects on proliferating cell nuclear antigen (PCNA) and activated caspase 3 (a-CASP3) protein availability in decidualized and antigestagen-treated DUS cells. Optical densities of PCNA (29 kDa) and a-CASP3 (20 kDa) were normalized against ACTB (45 kDa). One-way ANOVA was applied, revealing: *p* = 0.003 and *p* < 0.0001 for PCNA in response to mifepristone or aglepristone, respectively; *p* = 0.01 and *p* < 0.0001 for a-CASP3 in response to mifepristone or aglepristone, respectively. The ANOVA tests were followed by Tukey-Kramer multiple-comparisons test. Numerical data are presented as mean ± standard deviation (SD). Bars with different asterisks differ at: (*) *p* < 0.05, (**) *p* < 0.01, (***) *p* < 0.001.

The effects of antigestagens on the expression of proliferation marker PCNA and of the pro-apoptotic marker a-CASP3 (activated caspase 3) were assessed in experiments employing mifepristone and aglepristone. Both functional markers, PCNA and a-CASP3, were significantly modulated (the statistical details are presented in Figure 6 (the whole blot figure is presented in the Supplementary File S1)) in response to the treatment with both antigestagens, but in opposite regulatory directions. Accordingly, whereas the protein levels of PCNA decreased strongly, a-CASP3 was significantly elevated in response to the treatment. In both cases, the strongest effects were observed in response to the highest dosages of antigestagens.

## 4. Discussion

The decreased signaling of P4 is a key event in the termination of pregnancy. Accordingly, in the dog, either the decreased availability of circulating P4 closer to term, or the functional blockage of the P4 response with antigestagens, results in parturition or abortion [13,21,35]. Consequently, due to the importance of decidual cells for the maintenance of pregnancy, understanding the effects of P4/PGR signaling in these cells is crucial to unveiling the mechanisms of canine parturition.

Here, to gain initial insights into PGR-mediated effects in decidual cells, a well-characterized model of canine decidualization with DUS cells was employed [3,9,25]. Type II antigestagens were used to interfere with PGR-signaling in decidualized DUS cells, and then the effects on the cells’ decidualization features were assessed. The morpho-functional changes observed in decidualized DUS cells, as well as the increased expression of transcripts encoding for the major decidualization markers, i.e., *IGF1*, *PTGES*, *PRLR* and *PGR*, were in agreement with the previous findings [3,9]. Similarly, the increased expression of *ECM1*, *COL4* and *CX43*, as well as downregulation of *IGF2*, followed the previously described patterns [25], cumulatively serving as positive controls for cells attaining the mesenchymal-epithelial transition characteristic of decidualization [25].

In time-course experiments, no apparent effects of the treatment on the morphology of DUS cells were noticed, and both vimentin and pSV40 were continuously expressed. Previous studies reported that treatments with up to 10 μΜ mifepristone for at least 24 h did not have significant cytotoxic effects on different cell lines, e.g., human endometrial endothelial cells (HEECs) or the ovarian cancer cell lines SK-OV-3 and OV2008 [36,37]. Thus, using dosages of antigestagens between 0.5–2 μM in the present approach appeared appropriate.

The inhibition of PGR-signaling in decidualized DUS cells with both type II antigestagens resulted in suppressed expression of all markers induced during decidualization; the already lowered expression of *IGF2* remained unaffected. Contrasting with the human model, P4 does not induce spontaneous decidualization in vivo in the dog [3,6,7]. Nevertheless, as shown in vitro, P4 does have a decidualization capacity in the dog, modulating the expression of decidualization markers, e.g., *PRLR* and *PGR* [25]. PGE2 can also induce the decidualization of DUS cells, upregulating, e.g., *PRLR*, *PGR* and *PTGES*, through its cAMP-mediating PGE2 receptors PTGER2 and -4 [25]. P4 and PGE2, at least to some extent, appear to work independently, with P4, but not PGE2, downregulating *IGF2* expression, whereas PGE2 can upregulate *IGF1* independently of P4 [25]. Nevertheless, a PGE2/P4 interplay does appear to be present in canine decidual cells, with P4 modulating PGE2-mediated effects in DUS cells by increasing *PTGER2* transcriptional availability and downregulating *PTGER4* [25]. This regulatory loop between P4 and PGE2 is further supported by the suppressive effects of PGR blockers exerted on *PTGES* expression. A similar interplay between P4 and PGE2 also appears to be present in the human model, with PGE2-induced decidualization depending on P4-dependent expression of PTGER2 and -4 expression [38]. This also suggests that the decreased P4 signaling observed at parturition not only directly affects P4-responsive factors, but indirectly dysregulates PGE2-mediated effects in decidual cells.

It is noteworthy that antigestagens suppressed *PRLR* expression, an important decidualization marker displaying high expression, both in vivo [30] and in vitro [3,9]. Additionally, both aglepristone and mifepristone downregulated *IGF1*, implying the involvement of PGR in the regulation of the availability of this growth factor. It should be mentioned, however, that type II antigestagens actively induce negative effects on PGR-target genes by recruiting transcriptional repressors [19,20]. Thus, it is possible that the observed suppressive effects on target gene expression are due to transdominant suppressive effects of type II antigestagens and are not limited to the disruption of PGR signaling. At the transcriptional level, mifepristone appeared to reveal stronger effects upon mRNA abundance of some of the investigated factors. This is evident when, e.g., the expression of PGR is considered. Thus, mifepristone, but not aglepristone, decreased the expression of *PGR* mRNA in decidualized DUS cells. This is an interesting finding indicating possible differences in the functionality of the two antigestagens. Accordingly, in the as-yet unpublished results, aglepristone and mifepristone differed in their transcriptomic and kinomic effects exerted upon decidualized DUS cells, with kinomics being a merger between genomics and proteomics. On the other side, and particularly when used at higher dosages, both antigestagens significantly reduced the expression of the respective PGR protein. Whether these effects involve additional indirect, and/or posttranscriptional regulatory mechanisms, remains to be investigated. It needs to be added, that the presence and function of the two possible different PGR isoforms identified in other species, e.g., humans (90 kDa PGR-A and 120 kDA PGR-B), has not yet been distinguished in the utero-placental structures of the dog. The antibody used here is directed against a recombinant protein corresponding to amino acids 922–933 of human PGR. As shown in Figure 2, the clearly detectable bands in presented immunoblots performed with proteins isolated from DUS cells were recognized as proteins of approximately 100 kDa.

Nevertheless, despite aglepristone being a chemical derivative of mifepristone [21] and both antigestagens presenting similar mechanisms of action and higher affinity to PGR than P4 [23,39], the effects exerted by aglepristone and mifepristone do not appear to be identical. This could be an interesting finding of clinical importance. Furthermore, the possibility of interaction with other receptors should not be excluded. In addition to PGR, mifepristone has a high affinity for the glucocorticoid receptor, and can also interact with the androgen receptor [39,40,41]. To the authors’ knowledge, no such information is available for aglepristone. Yet, in the canine placenta, the glucocorticoid receptor GR/NR3C1 appears to be predominant in fetal trophoblast cells, with decidual cells devoid of its detectable expression [42]. Moreover, the presence and role of membrane P4 receptors (i.e., PGRMC1, -2, mPRα, -β, -γ) in canine decidual cells, as well as the interaction of both type II antigestagens with these receptors, still need to be elucidated, as currently most of the utero-placental effects of antigestagens are attributed to their effects being exerted upon the nuclear PGR. Nevertheless, as a new finding from the present study, the suppressive effects of mifepristone and aglepristone on *PGR* expression suggest that the previously observed ability of P4 to regulate its own receptor in decidualized DUS cells [25] is, at least in part, mediated through PGR.

Following the observed modulation of decidualization markers, the authors took the opportunity to investigate other possible PGR-mediated effects in decidualized cells, represented by factors associated with the mesenchymal-epithelial transition of DUS cells. As for *PGR*, mifepristone, but not aglepristone, decreased the transcriptional availability of *COL4*. Despite the lack of a response at the protein level, this is an important observation as it emphasizes the contribution of PGR signaling to the morpho-functional transition of stromal cells underlying decidualization. It can be assumed that the time needed for protein degradation and turnover might be responsible for the lack of changes in COL4 protein levels. The same may account for the ECM1 expression, the transcriptional availability of which was suppressed in response to both antigestagens. Following this line, mifepristone treatment in vivo was associated with decreased COL4 availability around decidual cells of rhesus monkeys [43]. As for ECM1, its protective role, possibly in reducing trophoblast-derived metalloproteinase activity in the canine placenta, was suggested recently [25]. With regard to VIM and αSMA, decidualization was associated with a decreased expression of the latter, although neither was affected by antigestagen treatment and, as mentioned elsewhere, the cells retained their mesenchymal character.

Conversely, the expression of CX43 was strongly suppressed by both antigestagens, including diminished protein levels. As a gap junction molecule, CX43 is involved in the decidualization of uterine stromal cells in the dog and other species displaying invasive placentation [25,44,45]. Underlying its importance during the maintenance of pregnancy is the fact that blockage of CX43 functionality impairs the decidualization process and leads to apoptosis of stromal cells, as shown e.g., in humans [46,47]. This is also corroborated by the current findings showing suppressive effects of antigestagens on the expression of the proliferation marker PCNA, occurring concomitantly with increased availability of a-CASP3. Similarly, in rats, mifepristone was associated with decreased decidual expression of PCNA in vivo [48]. In human endometrial cells, mifepristone induced cell cycle arrest and stimulated a-CASP3-dependent apoptosis in vitro [49].

These apparent antiproliferative and/or pro-apoptotic activities of antigestagens in P4 responsive tissues might be responsible for the positive effects of the use of mifepristone in adjuvant therapy of some human disorders, e.g., breast cancer and endometriosis [50,51]. Accordingly, in dogs, positive effects of aglepristone in the clinical management of vaginal tumors and mammary carcinomas were reported, e.g., by decreasing their size [52,53,54]. Thus, in the placenta, a decreased viability of decidual cells, associated with the modulation of PCNA and a-CASP3, and disruption of cellular adhesion, as suggested by decreased expression of CX43, might represent important mechanisms in the antigestagen-induced termination of pregnancy.

## 5. Conclusions

**Figure 7:**
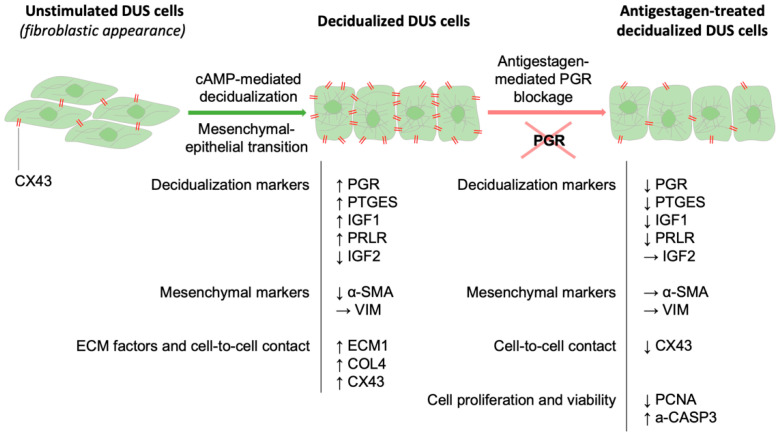
Schematic representation of the changes mediated by in vitro decidualization on DUS cells and the effects mediated by functional blocking of PGR with antigestagens. The main findings of the present study are summarised, with arrows indicating increased (↑), decreased (↓) or unaffected (→) expression. The fibroblastic appearance of DUS cells changed to a rounded shape after in vitro decidualization, which was unaffected by 6h treatment with antigestagens. Increased expression of decidualization markers, extracellular matrix (ECM) factors and CX43, was observed after cAMP-mediated decidualization. The mesenchymal-epithelial transition of the cells was associated with the cells retaining their mesenchymal character (VIM and α-SMA) and increasing COL4 availability (characteristic of basal lamina). The most important changes after treatment with antigestagens included decreased levels of important decidualization markers and of the gap junction molecule CX43. Additionally, blocking PGR induced antiproliferative and pro-apoptotic effects on decidualized DUS cell (PCNA and a-CASP3). The present results highlight the crucial role of P4 signaling in the physiology of decidual cells, further implying its critical role in the termination of canine pregnancy.

The most important findings of the present study are schematically summarized in Figure 7. Considering the function of type II antigestagens as transdominant repressors, their effects in canine decidualized cells indicate downstream targets of PGR. Those include, i.a., decidualization markers, PTGES, PRLR, PGR and IGF1. Consequently, supporting the authors’ working hypothesis, the withdrawal of PGR function suppressed their expression and even more strongly emphasized the importance of P4/PGR signaling in the maintenance of canine pregnancy. Importantly, as discussed elsewhere, despite their similarities, both antigestagens appear to differ in their pharmacological and biological effects in canine decidual cells. Together with the negative effects exerted upon CX43, the increased a-CASP3 expression associated with antiproliferative effects points towards the molecular mechanisms involved in the induction of canine parturition. In the placenta, decidual cells are embedded in fetal trophoblast and also directly communicate with maternal endothelial cells [55]. Thus, disruption of CX43 expression could affect the cell-to-cell interaction between decidual cells and their neighboring cellular compartments, leading to apoptosis, disruption of vascular function and increased activity of the immune system, as reported in previous transcriptomic study [24], and associated with the prepartum release of PGF2α [56,57,58]. In this context, the possible effects of antigestagens upon membrane-bound P4 receptors also require future attention. Cumulatively, while revealing possible regulatory mechanisms, present results strongly encourage future, more detailed studies on the antigestagen-mediated effects in the canine decidual cells playing key roles in the canine prepartum signaling cascade.

## Figures and Tables

**Table 1:** List of all TaqMan system used for the semi-quantitative RT-PCR.

Primer Gene Name	Accession Numbers	Primer Sequence		Product Length (bp)
*PTGES*	NM_001122854	Forward	5′-GTC CTG GCG CTG GTG AGT-3′	89
		Reverse	5′-ATG ACA GCC ACC ACG TAC ATC-3′	
		TaqMan probe	5′-TCC CAG CCT TCC TGC TCT GCA GC-3′	
*PRLR*	HQ267784	Forward	5′-GGA TCT TTG CCG TTC TTT-3′	92
		Reverse	5′-AAG GAT GCA GGT CAC CAT GCT AT-3′	
		TaqMan probe	5′-ATT ATG GTC GTA GCA GTG GCT TTG AAA GGC-3′	
*PGR*	NM_001003074	Forward	5′-CGA GTC ATT ACC TCA GAA GAT TTG TTT-3′	113
		Reverse	5′-CTT CCA TTG CCC TTT TAA AGA AGA-3′	
		TaqMan probe	5′-AAG CAT CAG GCT GTC ATT ATG GTG TCC TAA CTT-3′	
*ECM1*	XM_845921.4	Forward	5′-CAG TCT GGC TTC TCC CAC CTT A-3′	99
		Reverse	5′-GCG GTT TGT GTG GCT GTG A-3′	
		TaqMan probe	5′-AGA CTA GAT ATT CCC GCT GCT GCC GCT-3′	
*CX43*	AY462223	Forward	5-AAA AGA GAA CCC TGC CCT CAT C-3	91
		Reverse	5-AGG ACA CGA CCA GCA TGA AGA-3	
		TaqMan probe	5-ACT GCT TCC TCT CTC GCC CCA CG-3	
*IGF1*	NM_001313855	Applied Biosystems, prod nr. Cf02627846_m1		104
*IGF2*	NM_001195403	Applied Biosystems, prod nr. Cf02647136_m1		126
*COL4*	XM_022408226	Applied Biosystems, prod nr. Cf02696157_mH		82
*KDM4A*	XM_005629106	Applied Biosystems, prod nr. Cf02708629_m1		96
*EIF4H*	XM_014114129	Applied Biosystems, prod nr. Cf02713640_m1		136
*PTK2*	XM_005627993	Applied Biosystems, prod nr. Cf02684608_m1		104

**Table 2:** List of antibodies used for immunofluorescence (IF) staining and western blot (WB) analysis.

Antibody	Company	Reference Number	Host	Dilution
ECM1	Proteintech	11521-1-AP	Rabbit polyclonal	IF 1:100
COL4	Abcam	ab6586	Rabbit polyclonal	IF 1:300
CX43	Abcam	ab11370	Rabbit polyclonal	IF 1:400
SV40Tag	Abcam	ab16879	Mouse monoclonal	IF 1:500
Vimentin	Abcam	ab92574	Rabbit monoclonal	IF 1:500
				WB 1:400
Alexa fluor 488 goat anti-mouse IgG (H+L)	Invitrogen	A11029	Goat	IF 1:100
Alexa fluor 594 goat anti-rabbit IgG (H+L)	Invitrogen	A11037	Goat	IF 1:100
PGR	IOPath	IM1408-Clone PR10A9	Mouse monoclonal	WB 1:300
PCNA	Abcam	ab29	Mouse monoclonal	WB 1:1000
a-CASP3	BD Biosciences	559565	Rabbit monoclonal	WB 1:200
α-SMA	DAKO	GA61161-2	Mouse anti-human	WB 1:500
ACTB	Santa Cruz Biotechnology	sc-69879	Mouse monoclonal	WB 1:1000
Goat anti-mouse HRP-labelled secondary IgG	Promega	W402B	Goat anti-mouse IgG	WB 1:15,000
Goat anti-Rabbit IgG (H+L) Secondary Antibody, HRP	Thermo Fisher Scientific	31460	Goat anti-rabbit IgG	WB 1:15,000

## Data Availability

The data presented in this study are available on a reasonable request from the corresponding author.

## Supplementary Materials

The following supporting information can be downloaded at: https://www.mdpi.com/article/10.3390/ani12070798/s1, Supplementary File S1: The whole blot figure.

## References

[1] Kautz E., Gram A., Aslan S., Ay S.S., Selcuk M., Kanca H., Koldas E., Akal E., Karakas K., Findik M. (2014). Expression of genes involved in the embryo-maternal interaction in the early-pregnant canine uterus. Reproduction.

[2] Kowalewski M.P., Gram A., Kautz E., Graubner F.R. (2015). The Dog: Nonconformist, Not Only in Maternal Recognition Signaling. Adv. Anat. Embryol. Cell Biol..

[3] Graubner F.R., Reichler I.M., Rahman N.A., Payan-Carreira R., Boos A., Kowalewski M.P. (2017). Decidualization of the canine uterus: From early until late gestational in vivo morphological observations, and functional characterization of immortalized canine uterine stromal cell lines. Reprod. Domest. Anim..

[4] Okada H., Tsuzuki T., Murata H. (2018). Decidualization of the human endometrium. Reprod. Med. Biol..

[5] Bany B.M., Cross J.C. (2006). Post-implantation mouse conceptuses produce paracrine signals that regulate the uterine endometrium undergoing decidualization. Dev. Biol..

[6] Gellersen B., Brosens I.A., Brosens J.J. (2007). Decidualization of the human endometrium: Mechanisms, functions, and clinical perspectives. Semin. Reprod. Med..

[7] Graubner F.R., Gram A., Kautz E., Bauersachs S., Aslan S., Agaoglu A.R., Boos A., Kowalewski M.P. (2017). Uterine responses to early pre-attachment embryos in the domestic dog and comparisons with other domestic animal species. Biol. Reprod..

[8] Herington J.L., Bany B.M. (2009). Do molecular signals from the conceptus influence endometrium decidualization in rodents?. J. Exp. Zool. B Mol. Dev. Evol..

[9] Kautz E., de Carvalho Papa P., Reichler I.M., Gram A., Boos A., Kowalewski M.P. (2015). In vitro decidualisation of canine uterine stromal cells. Reprod. Biol.Endocrinol..

[10] Gram A., Boos A., Kowalewski M.P. (2014). Uterine and Placental Expression of Canine Oxytocin Receptor During Pregnancy and Normal and Induced Parturition. Reprod. Domest. Anim..

[11] Vermeirsch H., Simoens P., Hellemans A., Coryn M., Lauwers H. (2000). Immunohistochemical detection of progesterone receptors in the canine uterus and their relation to sex steroid hormone levels. Theriogenology.

[12] Vermeirsch H., Simoens P., Lauwers H. (2000). Immunohistochemical detection of the estrogen receptor-alpha and progesterone receptor in the canine pregnant uterus and placental labyrinth. Anat. Record..

[13] Kowalewski M.P., Beceriklisoy H.B., Pfarrer C., Aslan S., Kindahl H., Kucukaslan I., Hoffmann B. (2010). Canine placenta: A source of prepartal prostaglandins during normal and antiprogestin-induced parturition. Reproduction.

[14] Gram A., Fox B., Buchler U., Boos A., Hoffmann B., Kowalewski M.P. (2014). Canine placental prostaglandin E2 synthase: Expression, localization, and biological functions in providing substrates for prepartum PGF2alpha synthesis. Biol. Reprod..

[15] Gram A., Buchler U., Boos A., Hoffmann B., Kowalewski M.P. (2013). Biosynthesis and degradation of canine placental prostaglandins: Prepartum changes in expression and function of prostaglandin F2alpha-synthase (PGFS, AKR1C3) and 15-hydroxyprostaglandin dehydrogenase (HPGD). Biol. Reprod..

[16] Gram A., Hoffmann B., Boos A., Kowalewski M.P. (2015). Expression and localization of vascular endothelial growth factor A (VEGFA) and its two receptors (VEGFR1/FLT1 and VEGFR2/FLK1/KDR) in the canine corpus luteum and utero-placental compartments during pregnancy and at normal and induced parturition. Gen. Comp. Endocr..

[17] Leonhardt S.A., Edwards D.P. (2002). Mechanism of action of progesterone antagonists. Exp. Biol. Med..

[18] Beck C.A., Zhang Y., Weigel N.L., Edwards D.P. (1996). Two types of anti-progestins have distinct effects on site-specific phosphorylation of human progesterone receptor. J. Biol. Chem..

[19] Edwards D.P., Altmann M., DeMarzo A., Zhang Y., Weigel N.L., Beck C.A. (1995). Progesterone receptor and the mechanism of action of progesterone antagonists. J. Steroid Biochem. Mol. Biol..

[20] Klein-Hitpass L., Cato A.C., Henderson D., Ryffel G.U. (1991). Two types of antiprogestins identified by their differential action in transcriptionally active extracts from T47D cells. Nucleic Acids Res..

[21] Kowalewski M.P., Pereira M.T., Papa P., Gram A. (2020). Progesterone receptor blockers: Historical perspective, mode of function and insights into clinical and scientific applications. Tierarztl. Prax. Ausg. K Kleintiere Heimtiere.

[22] Baan M., Taverne M.A., Kooistra H.S., de Gier J., Dieleman S.J., Okkens A.C. (2005). Induction of parturition in the bitch with the progesterone-receptor blocker aglepristone. Theriogenology.

[23] Gogny A., Fieni F. (2016). Aglepristone: A review on its clinical use in animals. Theriogenology.

[24] Nowak M., Rehrauer H., Ay S.S., Findik M., Boos A., Kautz E., Kowalewski M.P. (2019). Gene expression profiling of the canine placenta during normal and antigestagen-induced luteolysis. Gen. Comp. Endocrinol..

[25] Graubner F.R., Pereira M.T., Boos A., Kowalewski M.P. (2020). Canine decidualization in vitro: Extracellular matrix modification, progesterone mediated effects and selective blocking of prostaglandin E2 receptors. J. Reprod. Dev..

[26] Owusu-Akyaw A., Krishnamoorthy K., Goldsmith L.T., Morelli S.S. (2019). The role of mesenchymal-epithelial transition in endometrial function. Hum. Reprod. Update.

[27] Zhang X.H., Liang X., Liang X.H., Wang T.S., Qi Q.R., Deng W.B., Sha A.G., Yang Z.M. (2013). The mesenchymal-epithelial transition during in vitro decidualization. Reprod. Sci..

[28] Chan J.W., Teo A.K.K. (2020). Replicates in stem cell models-How complicated!. Stem Cells.

[29] Kowalewski M.P., Schuler G., Taubert A., Engel E., Hoffmann B. (2006). Expression of cyclooxygenase 1 and 2 in the canine corpus luteum during diestrus. Theriogenology.

[30] Kowalewski M.P., Michel E., Gram A., Boos A., Guscetti F., Hoffmann B., Aslan S., Reichler I. (2011). Luteal and placental function in the bitch: Spatio-temporal changes in prolactin receptor (PRLr) expression at dioestrus, pregnancy and normal and induced parturition. Reprod. Biol. Endocrinol..

[31] Kowalewski M.P., Meyer A., Hoffmann B., Aslan S., Boos A. (2011). Expression and functional implications of peroxisome proliferator-activated receptor gamma (PPARgamma) in canine reproductive tissues during normal pregnancy and parturition and at antiprogestin induced abortion. Theriogenology.

[32] Nowak M., Aslan S., Kowalewski M.P. (2020). Determination of novel reference genes for improving gene expression data normalization in selected canine reproductive tissues—A multistudy analysis. BMC Vet. Res..

[33] Kowalewski M.P., Fox B., Gram A., Boos A., Reichler I. (2013). Prostaglandin E2 functions as a luteotrophic factor in the dog. Reproduction.

[34] Carpenter A.E., Jones T.R., Lamprecht M.R., Clarke C., Kang I.H., Friman O., Guertin D.A., Chang J.H., Lindquist R.A., Moffat J. (2006). CellProfiler: Image analysis software for identifying and quantifying cell phenotypes. Genome Biol..

[35] Kowalewski M.P., Tavares Pereira M., Kazemian A. (2020). Canine conceptus-maternal communication during maintenance and termination of pregnancy, including the role of species-specific decidualization. Theriogenology.

[36] Helmestam M., Lindgren K.E., Stavreus-Evers A., Olovsson M. (2014). Mifepristone-exposured human endometrial endothelial cells in vitro. Reprod. Sci..

[37] Goyeneche A.A., Caron R.W., Telleria C.M. (2007). Mifepristone inhibits ovarian cancer cell growth in vitro and in vivo. Clin. Cancer Res..

[38] Stadtmauer D.J., Wagner G.P. (2021). Single-cell analysis of prostaglandin E2-induced human decidual cell in vitro differentiation: A minimal ancestral deciduogenic signal. Biol. Reprod..

[39] Heikinheimo O., Kontula K., Croxatto H., Spitz I., Luukkainen T., Lahteenmaki P. (1987). Plasma concentrations and receptor binding of RU 486 and its metabolites in humans. J. Steroid Biochem..

[40] Gagne D., Pons M., Philibert D. (1985). RU 38486: A potent antiglucocorticoid in vitro and in vivo. J. Steroid Biochem..

[41] Hoffmann B., Schuler G. (2000). Receptor blockers—General aspects with respect to their use in domestic animal reproduction. Anim. Reprod. Sci..

[42] Gram A., Trachsel A., Boos A., Kowalewski M.P. (2016). Elevated utero/placental GR/NR3C1 is not required for the induction of parturition in the dog. Reproduction.

[43] Pan Y., Liu Z., Zhu C. (2004). Effect of mifepristone on the expression of chorionic gonadotropin beta subunit and collagen type IV in female rhesus monkey decidua and villus at early gestation. Zhonghua Nan Ke Xue.

[44] Laws M.J., Taylor R.N., Sidell N., DeMayo F.J., Lydon J.P., Gutstein D.E., Bagchi M.K., Bagchi I.C. (2008). Gap junction communication between uterine stromal cells plays a critical role in pregnancy-associated neovascularization and embryo survival. Development.

[45] Orlando-Mathur C.E., Bechberger J.F., Goldberg G.S., Naus C.C., Kidder G.M., Kennedy T.G. (1996). Rat endometrial stromal cells express the gap junction genes connexins 26 and 43 and form functional gap junctions during in vitro decidualization. Biol. Reprod..

[46] Yu J., Wu J., Bagchi I.C., Bagchi M.K., Sidell N., Taylor R.N. (2011). Disruption of gap junctions reduces biomarkers of decidualization and angiogenesis and increases inflammatory mediators in human endometrial stromal cell cultures. Mol. Cell Endocrinol..

[47] Yu J., Berga S.L., Zou W., Sun H.Y., Johnston-MacAnanny E., Yalcinkaya T., Sidell N., Bagchi I.C., Bagchi M.K., Taylor R.N. (2014). Gap junction blockade induces apoptosis in human endometrial stromal cells. Mol. Reprod. Dev..

[48] Ogle T.F., George P., Dai D. (1998). Progesterone and estrogen regulation of rat decidual cell expression of proliferating cell nuclear antigen. Biol. Reprod..

[49] Li A., Felix J.C., Minoo P., Amezcua C.A., Jain J.K. (2005). Effect of mifepristone on proliferation and apoptosis of Ishikawa endometrial adenocarcinoma cells. Fertil. Steril..

[50] Giulianelli S., Molinolo A., Lanari C. (2013). Targeting Progesterone Receptors in Breast Cancer. Vitam. Horm..

[51] Khaled Z., Serdar E.B. (2003). Encyclopedia of Hormones.

[52] Rollon E., Millan Y., de las Mulas J.M. (2008). Effects of aglepristone, a progesterone receptor antagonist, in a dog with a vaginal fibroma. J. Small Anim. Pract..

[53] Guil-Luna S., Millan Y., De Andres J., Rollon E., Domingo V., Garcia-Macias J., Sanchez-Cespedes R., Martin de Las Mulas J. (2017). Prognostic impact of neoadjuvant aglepristone treatment in clinicopathological parameters of progesterone receptor-positive canine mammary carcinomas. Vet. Comp. Oncol..

[54] Pieczewska B., Glinska-Suchocka K., Nizanski W., Dzieciol M. (2021). Decreased Size of Mammary Tumors Caused by Preoperative Treatment with Aglepristone in Female Domestic Dogs (Canis familiaris) Do Not Influence the Density of the Benign Neoplastic Tissue Measured Using Shear Wave Elastography Technique. Animals.

[55] Kowalewski M.P., Kazemian A., Klisch K., Gysin T., Tavares Pereira M., Gram A. (2021). Canine Endotheliochorial Placenta: Morpho-Functional Aspects. Adv. Anat. Embryol. Cell Biol..

[56] Kowalewski M.P. (2012). Endocrine and molecular control of luteal and placental function in dogs: A review. Reprod. Domest. Anim..

[57] Nohr B., Hoffmann B., Steinetz B.E. (1993). Investigation of the endocrine control of parturition in the dog by application of an antigestagen. J. Reprod Fertil. Suppl..

[58] Baan M., Taverne M.A., de Gier J., Kooistra H.S., Kindahl H., Dieleman S.J., Okkens A.C. (2008). Hormonal changes in spontaneous and aglepristone-induced parturition in dogs. Theriogenology.

