# Supplementary material for: Antigestagens Mediate the Expression of Decidualization Markers, Extracellular Matrix Factors and Connexin 43 in Decidualized Dog Uterine Stromal (DUS) Cells
Source: Animals (Basel). 2022 Mar 22;12(7):798. doi: 10.3390/ani12070798 (PMC8996927; doi:10.3390/ani12070798)

Supplementary File S1

Mifepristone, PGR, approx. 100 kDa

(Cropped)

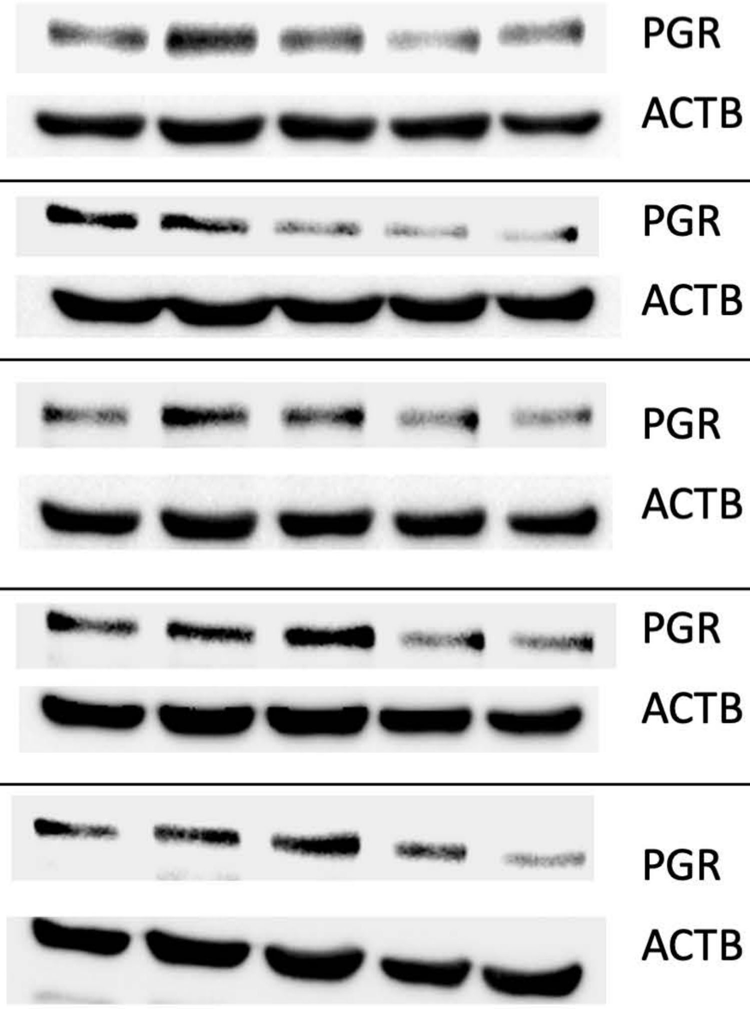

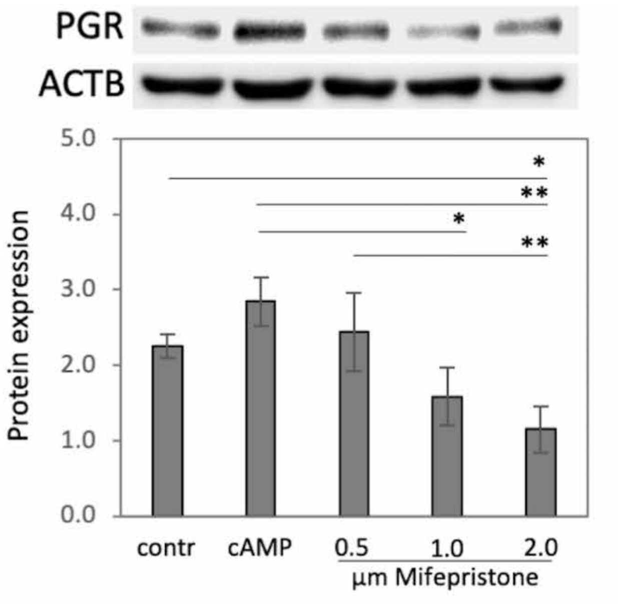

Aglepristone, PGR, approx. 100 kDa

(Cropped)

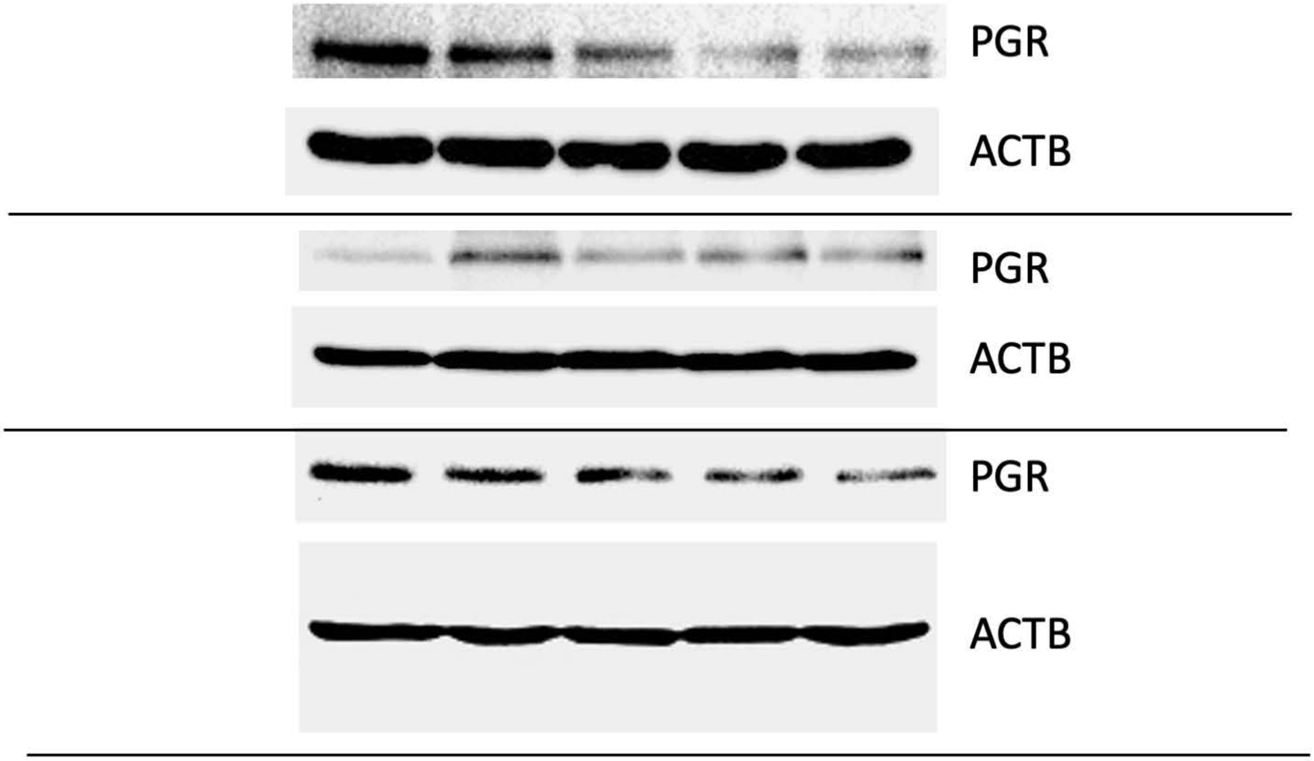

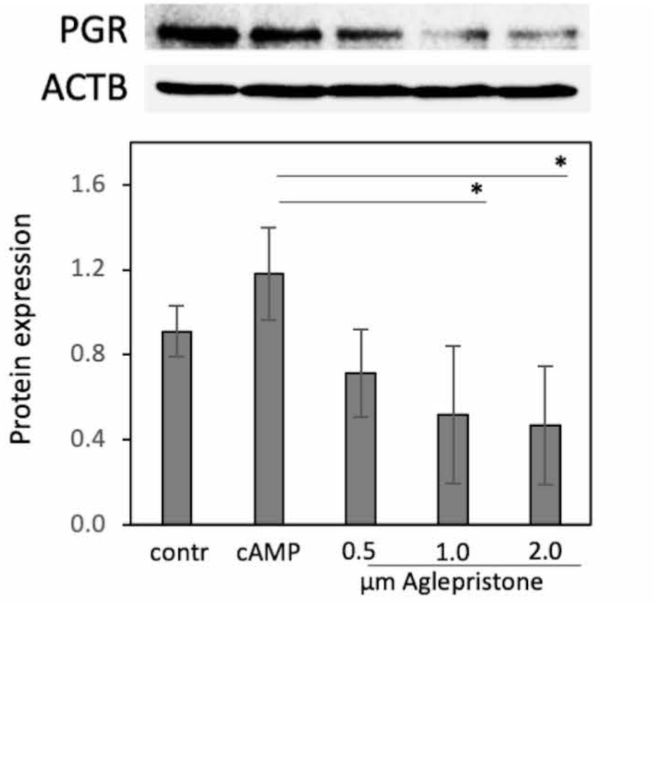

Mifepristone, VIM, 54 kDa

(Cropped)

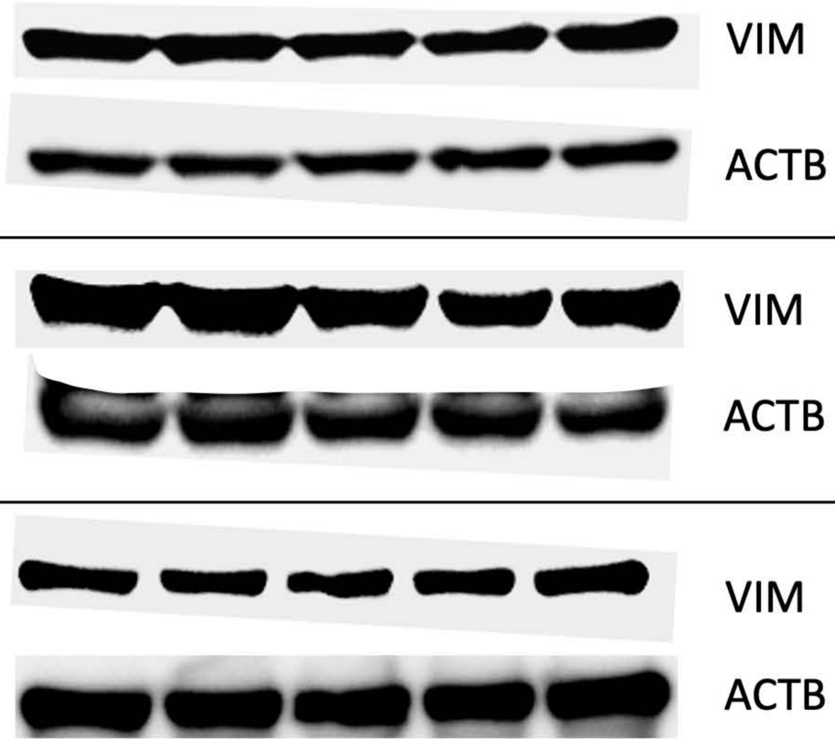

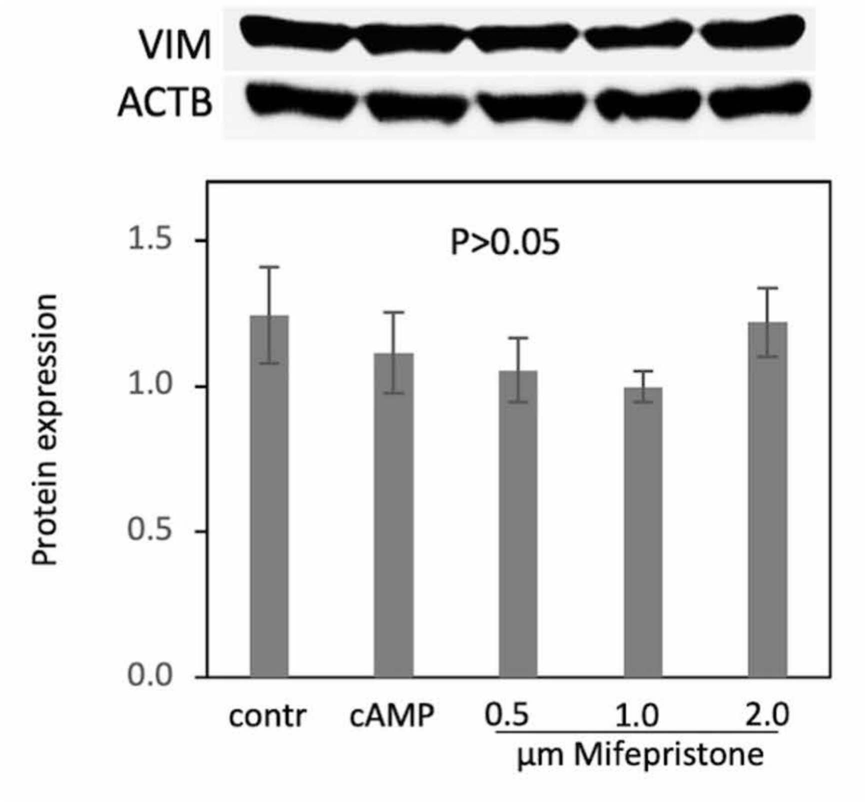

Aglepristone, VIM, 54 kDa

(Cropped)

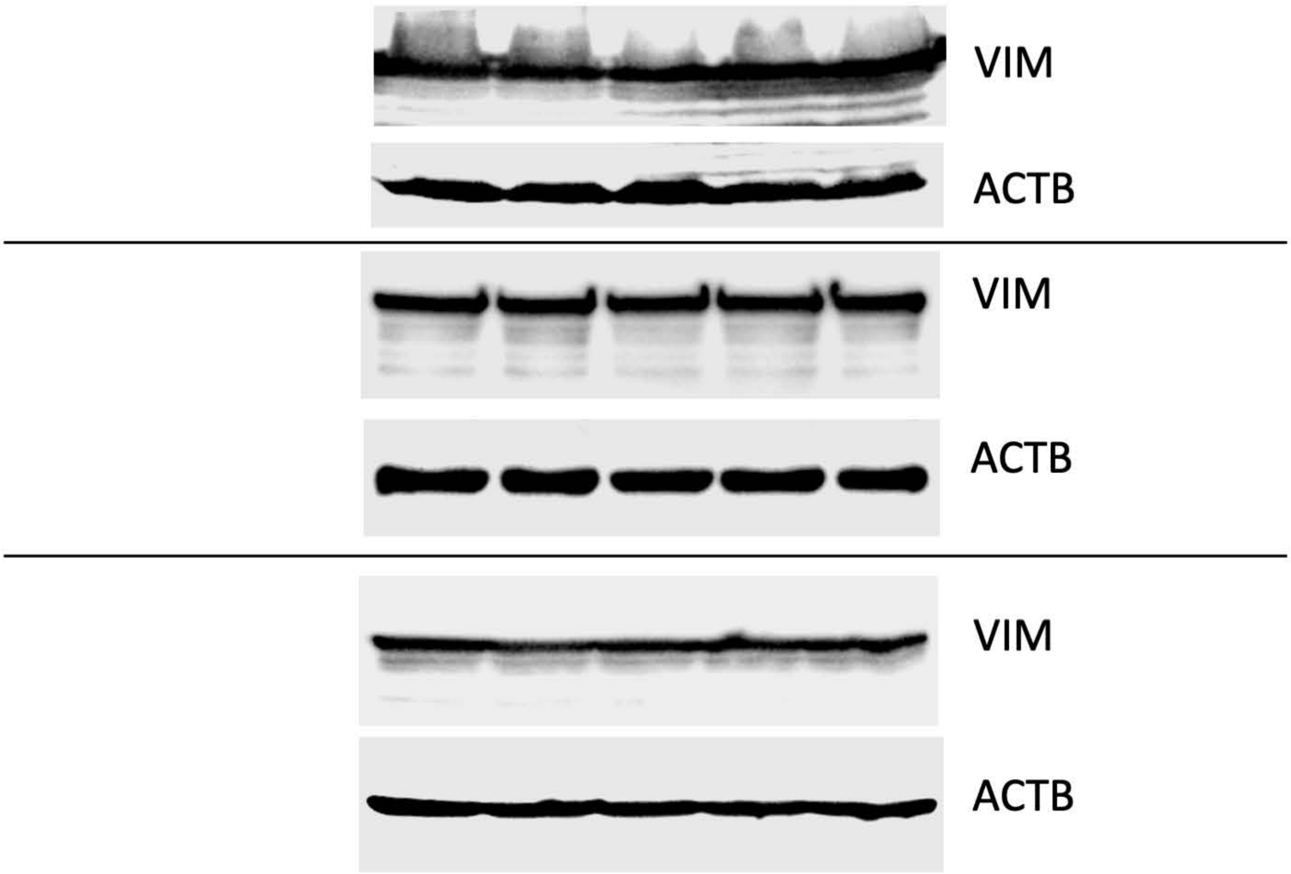

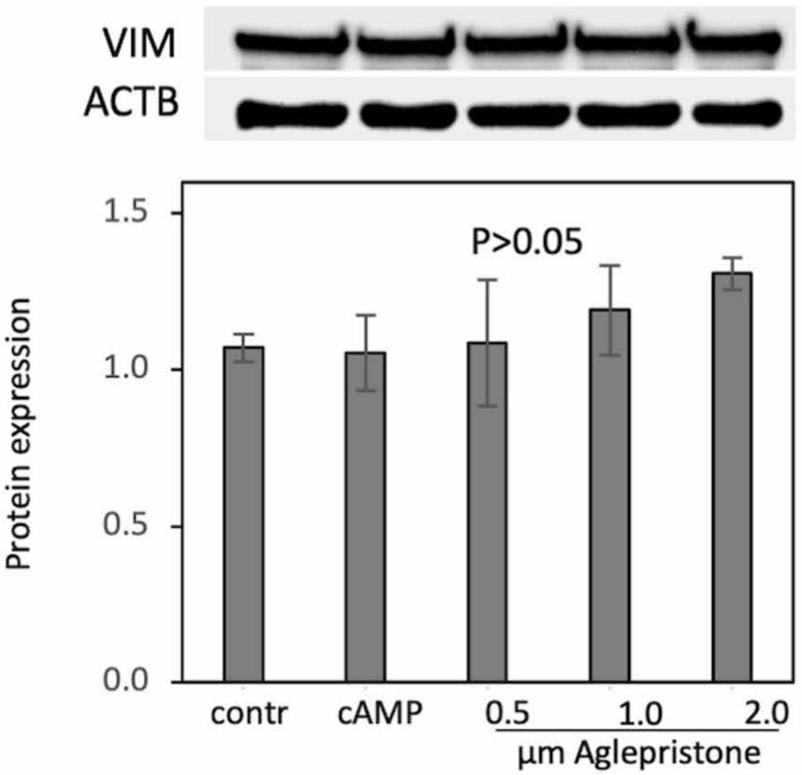

Mifepristone, α-SMA, 42 kDa

(Cropped)

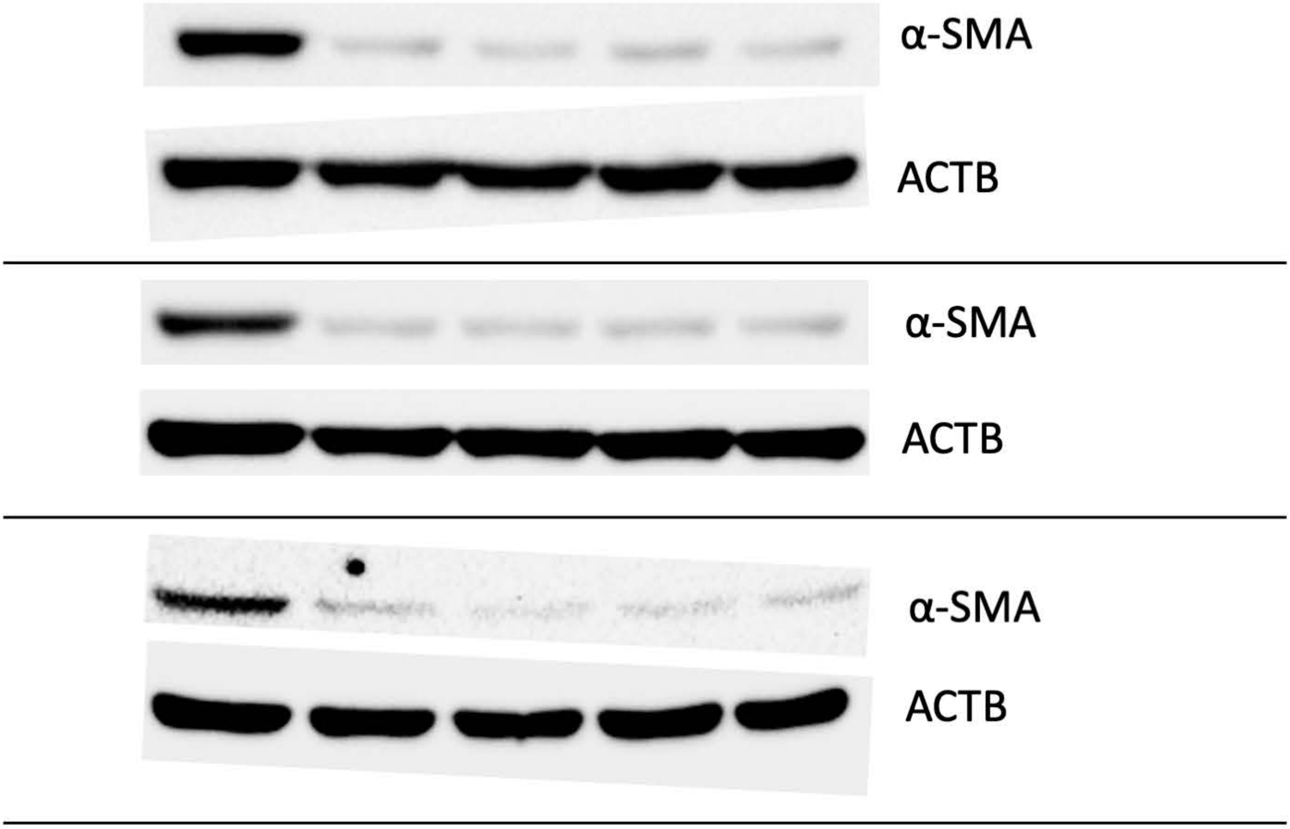

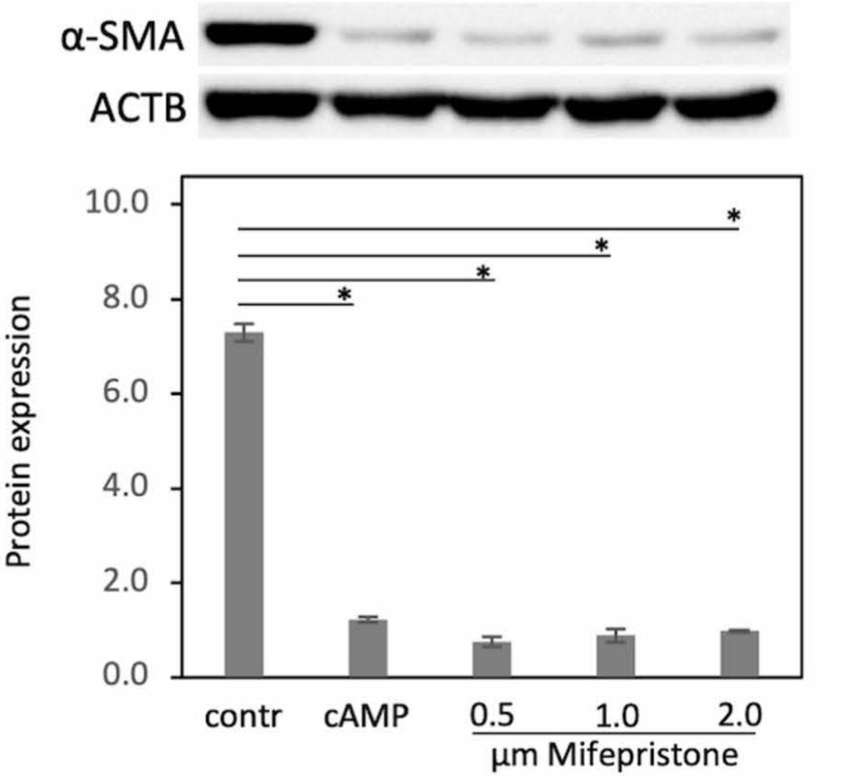

Aglepristone, α-SMA, 42 kDa

(Cropped)

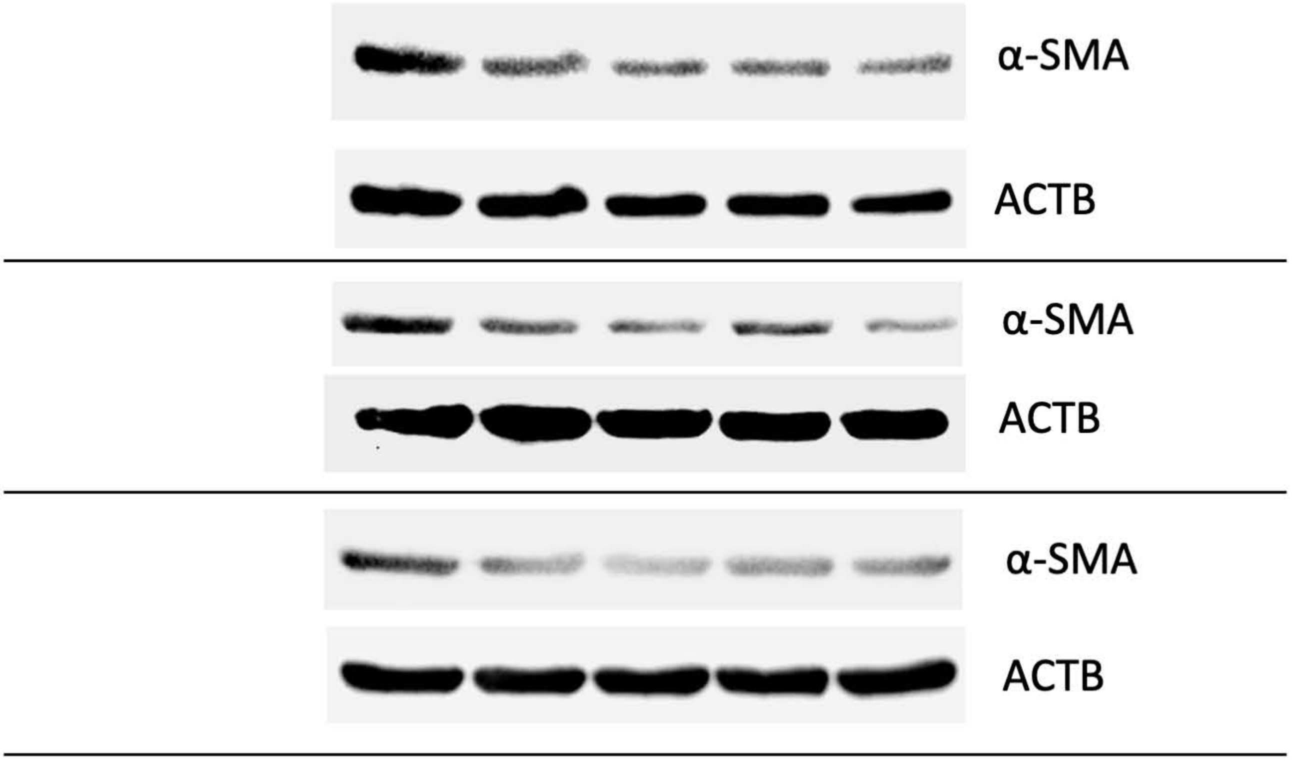

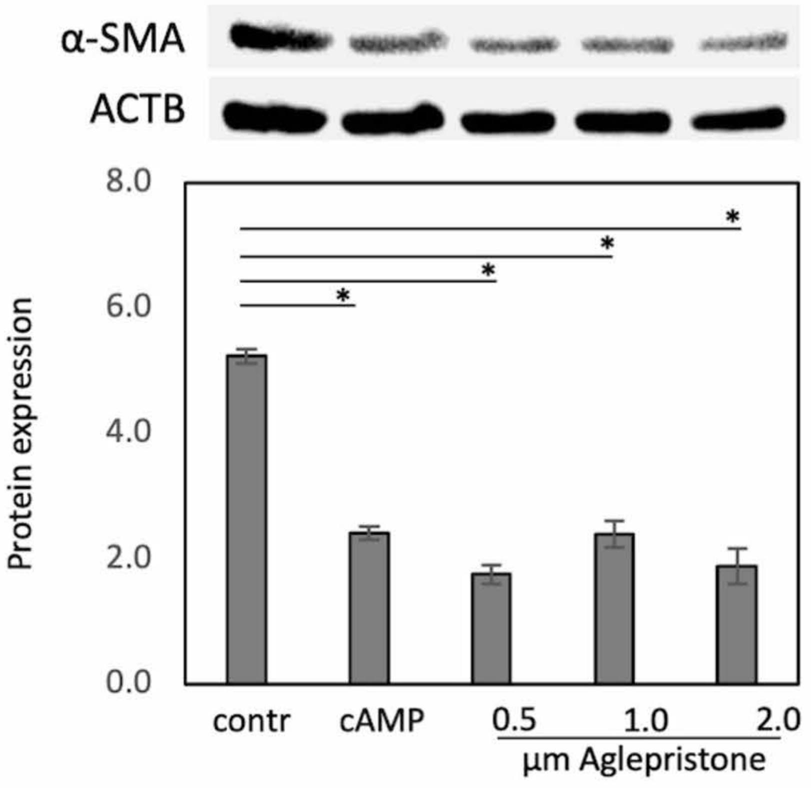

Mifepristone, PCNA, 29 kDa

(Cropped)

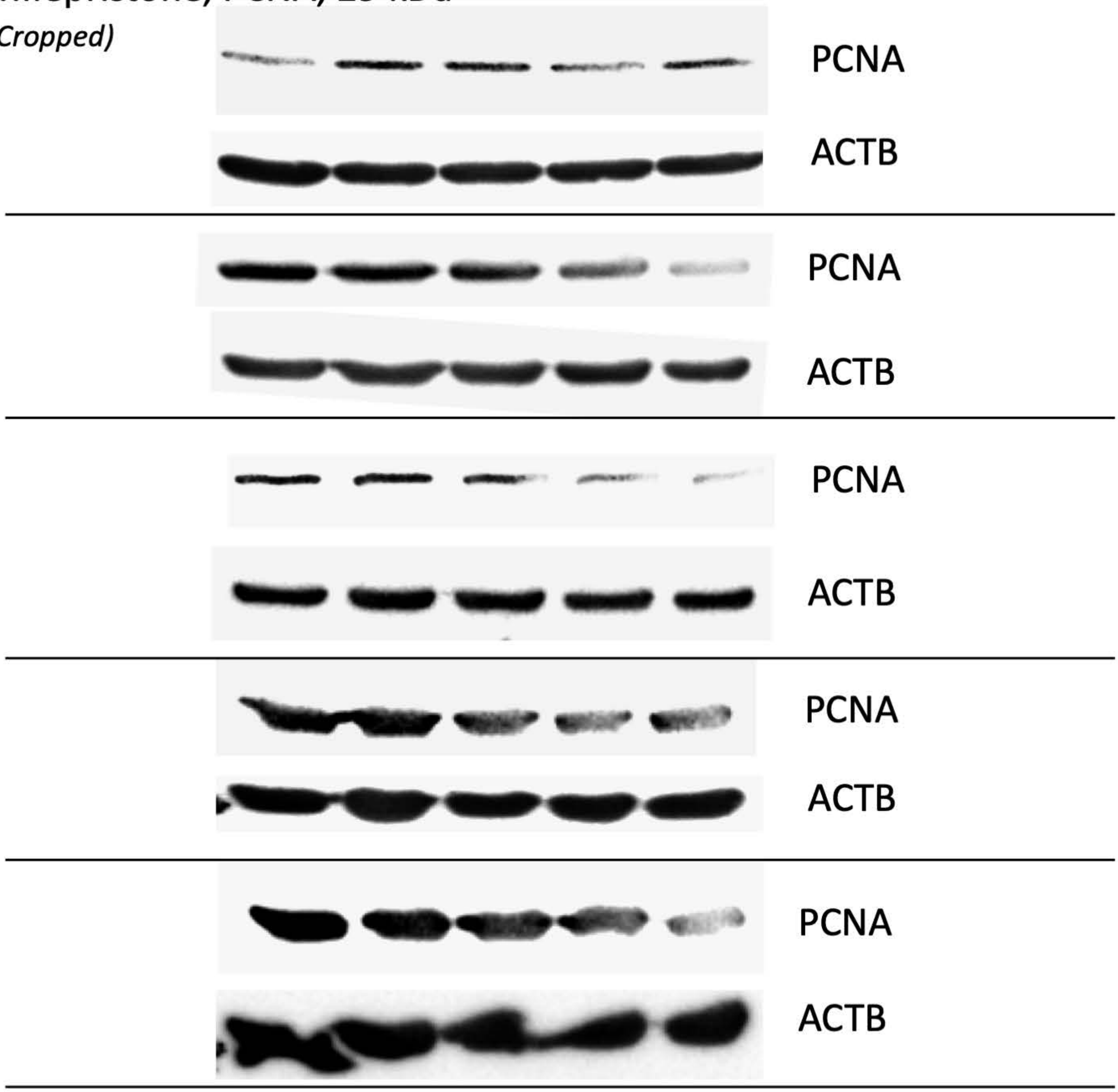

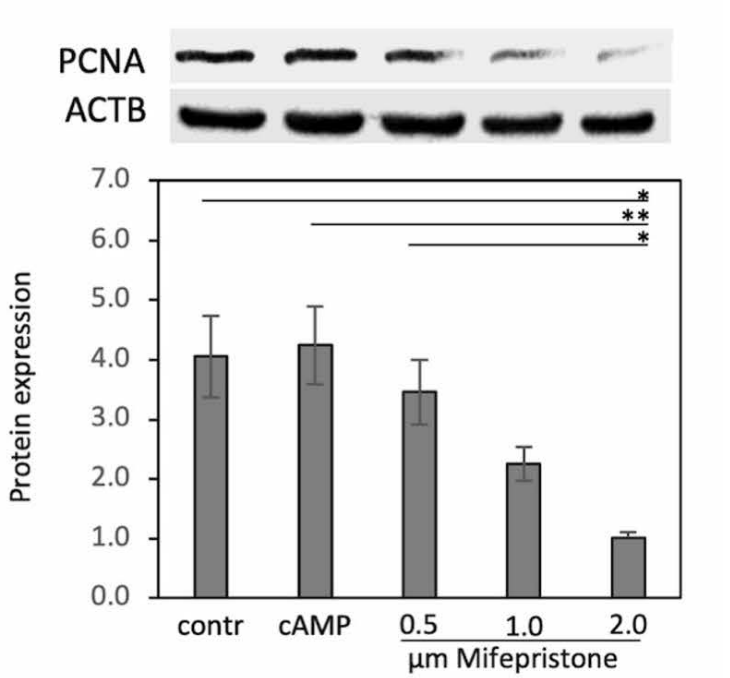

Aglepristone, PCNA, 29 kDa

(Cropped)

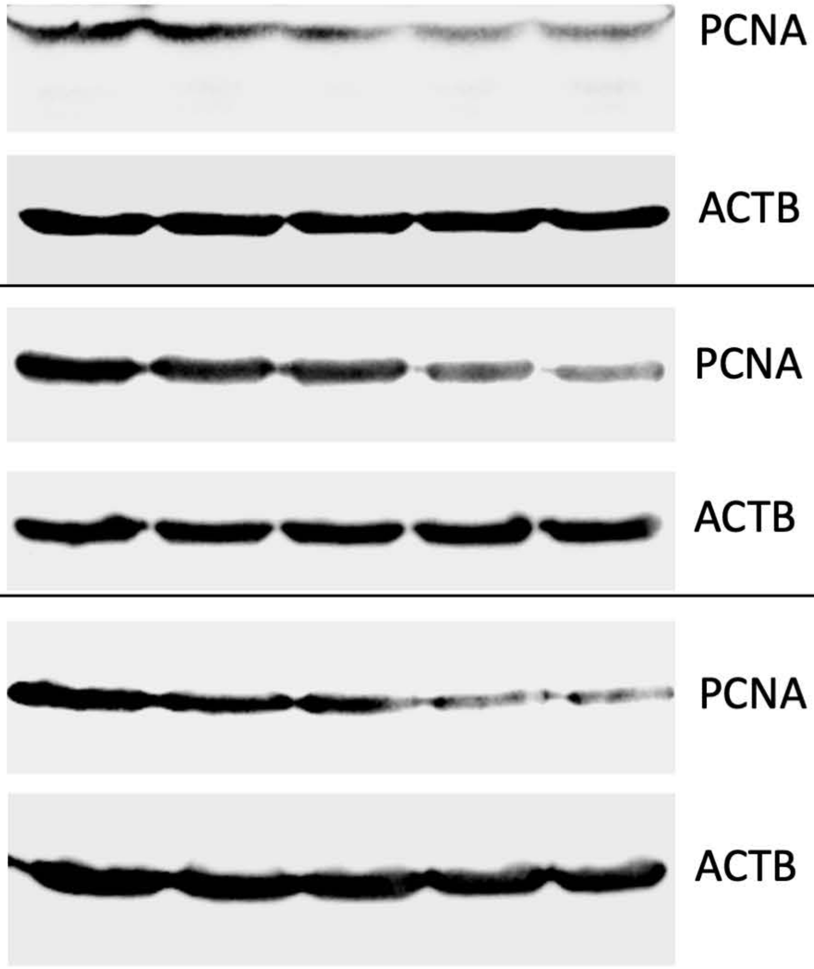

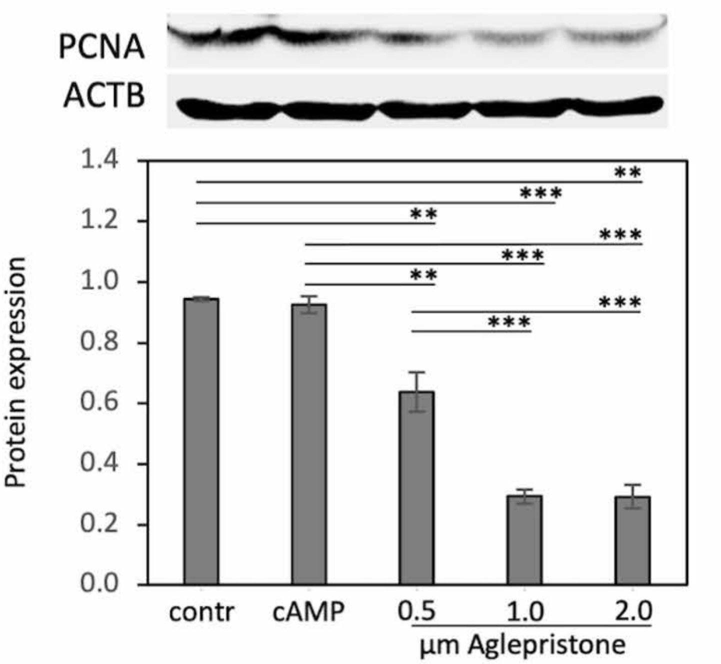

Mifepristone, a-CASP, 20 kDA

(Cropped)

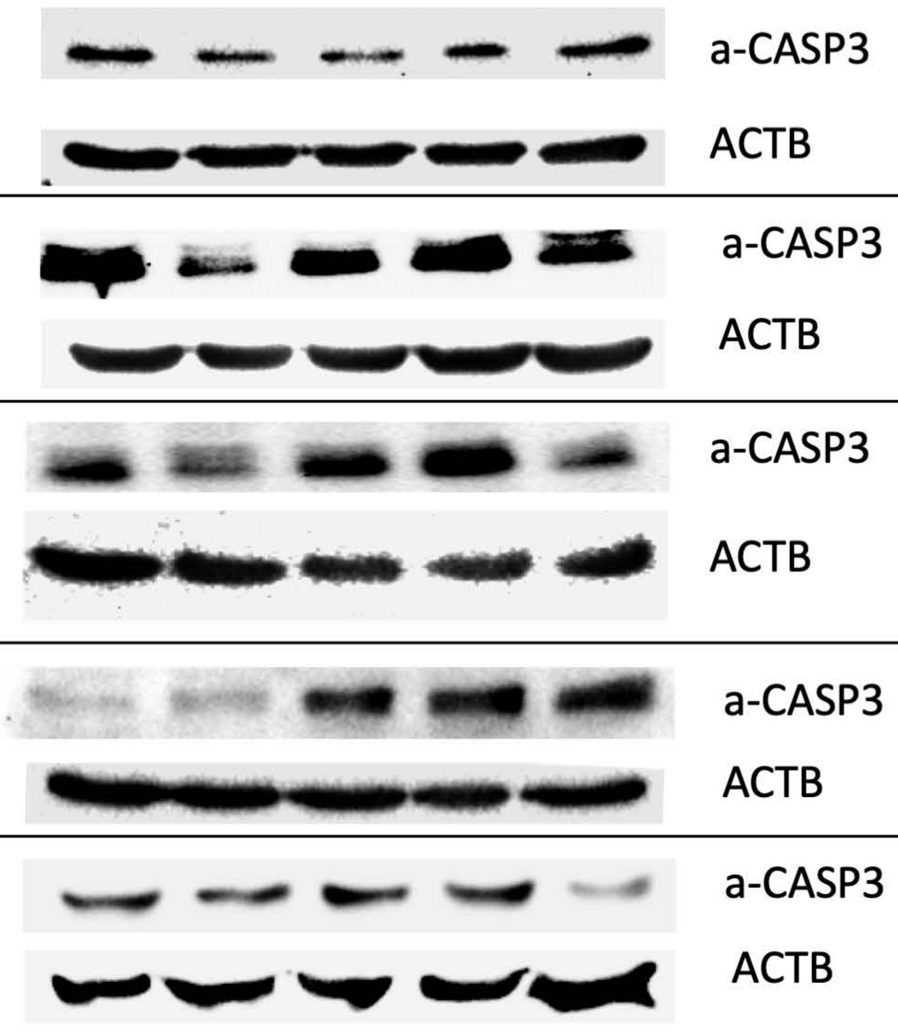

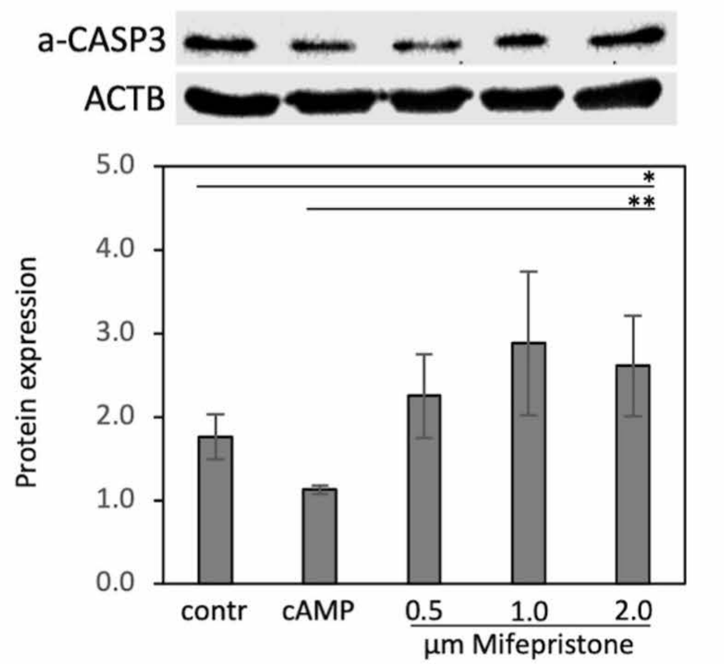

Aglepristone, a-CASP, 20 kDA

(Cropped)

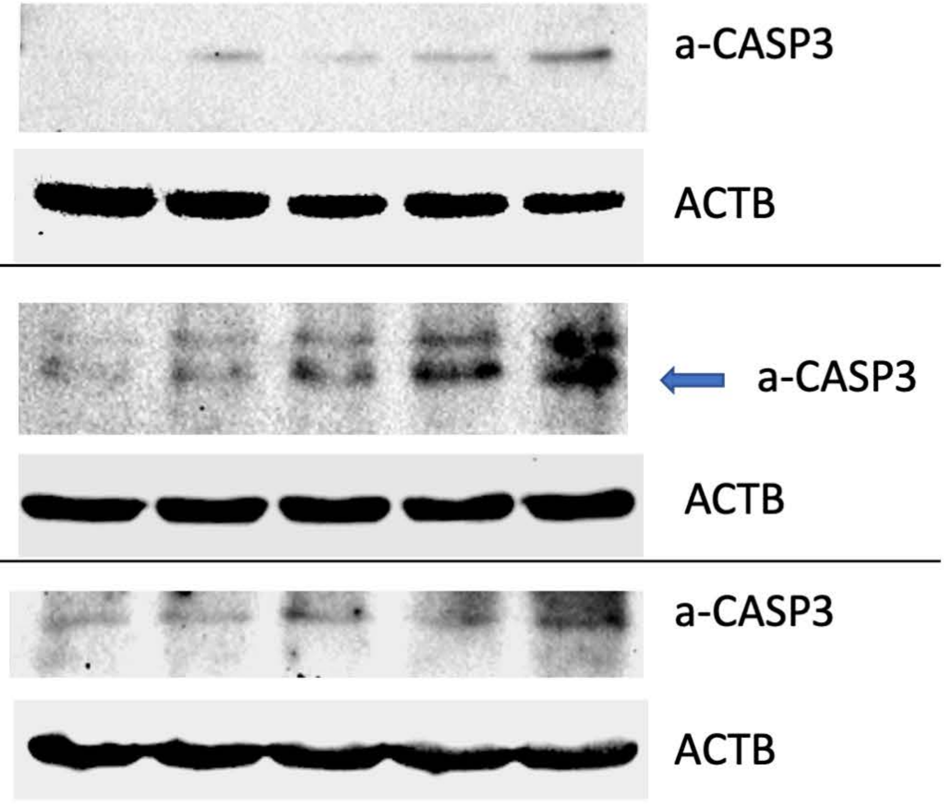

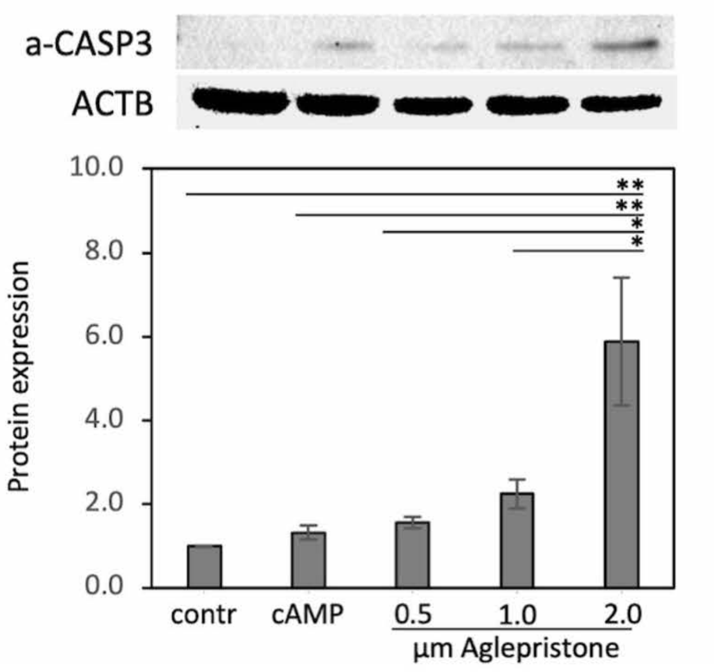

Mifepristone

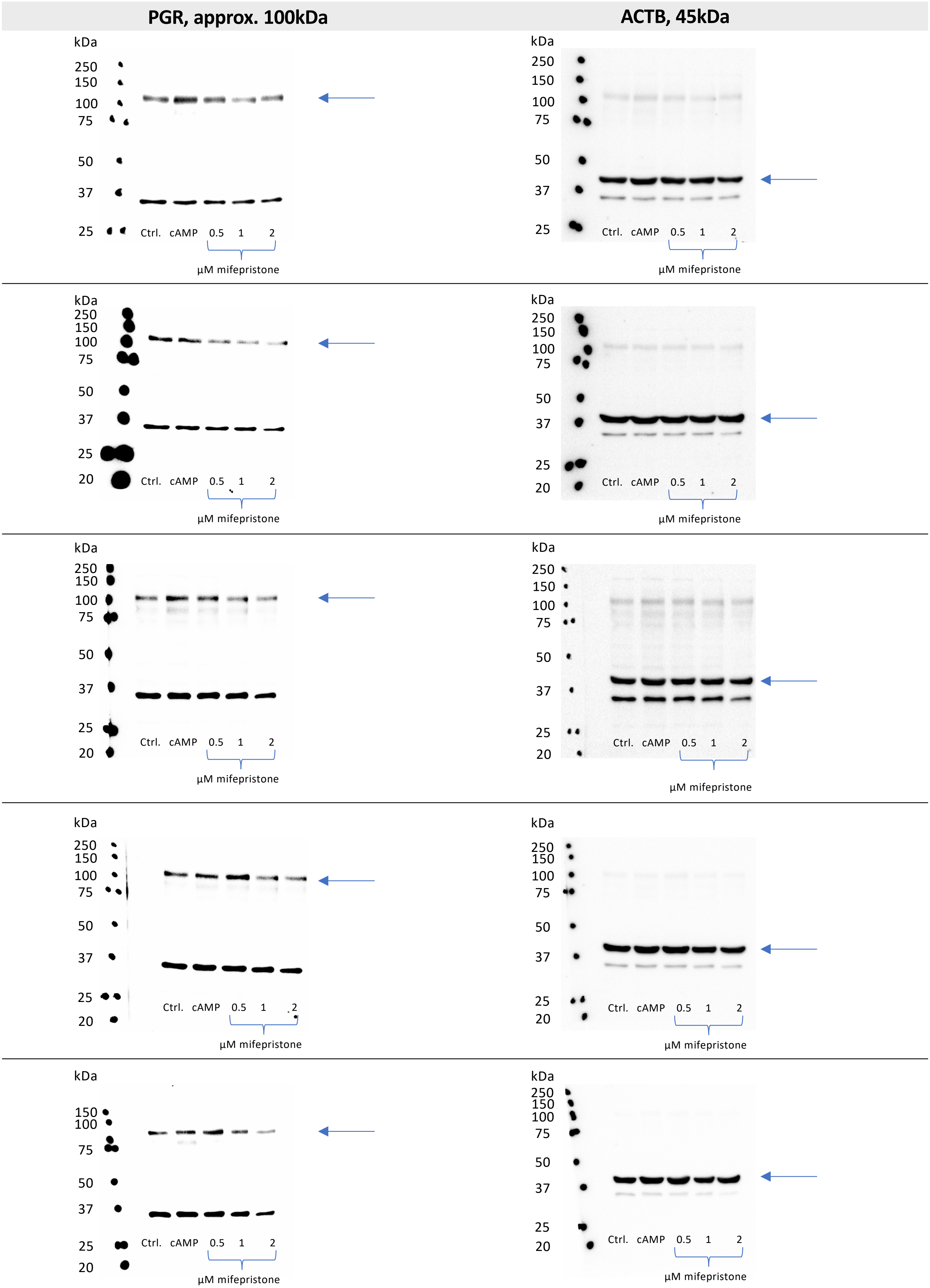

Aglepristone

PGR, approx. 100kDa	ACTB, 45kDa

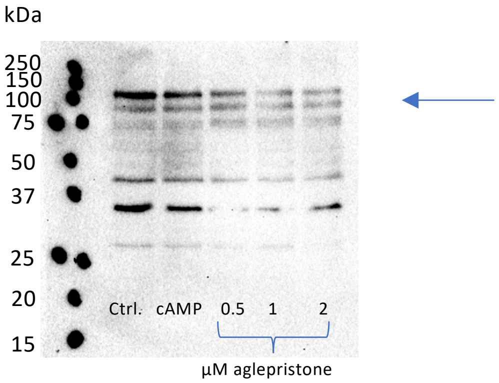

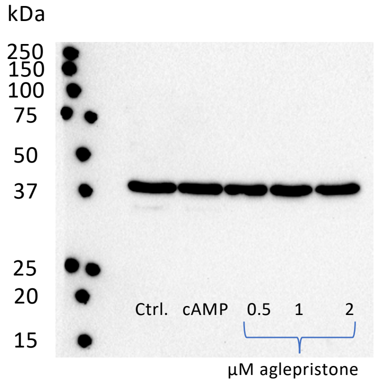

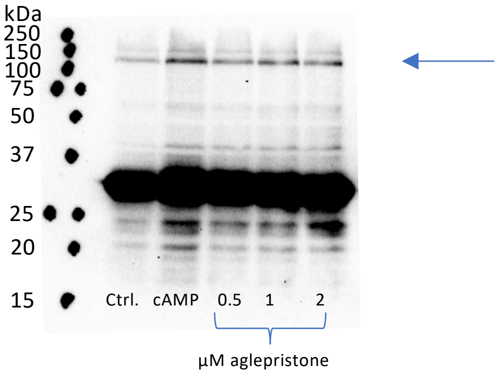

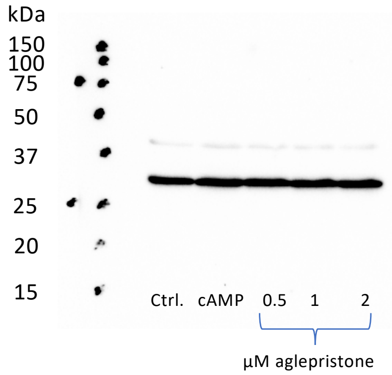

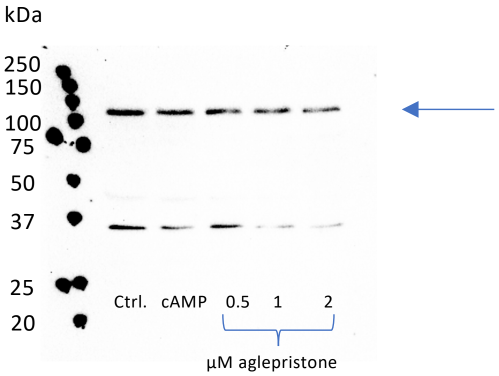

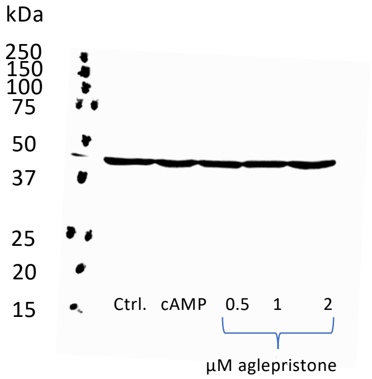

Mifepristone

VIM, 54kDa | **ACTB, 45kDa**

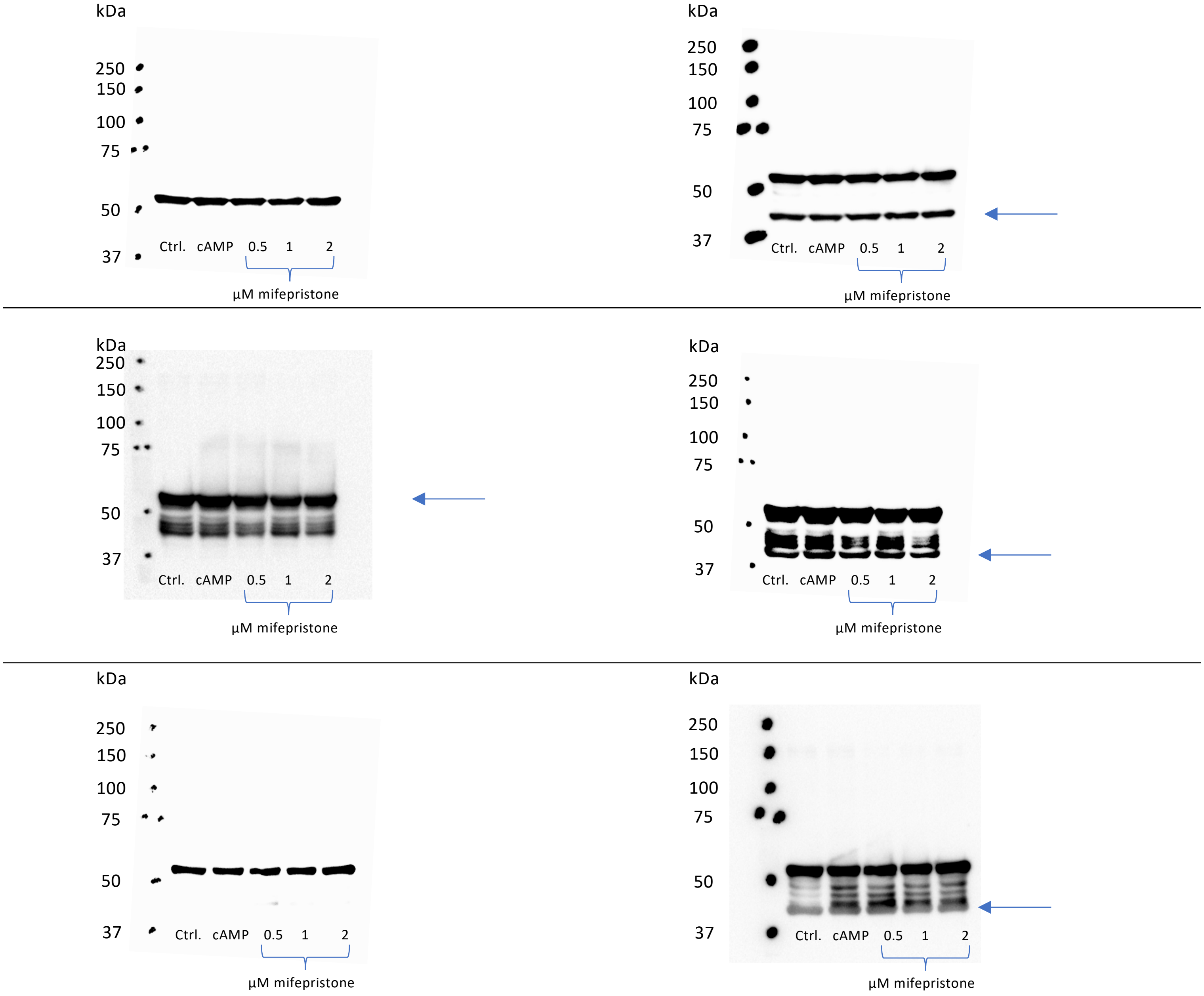

Aglepristone

VIM, 54kDa | **ACTB, 45kDa**

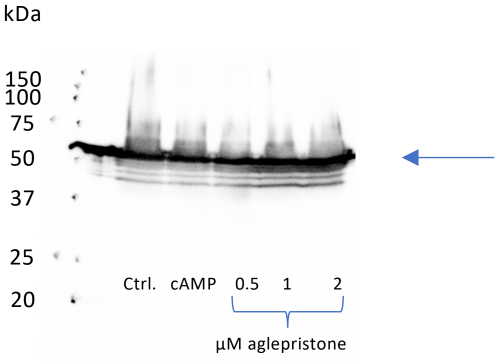

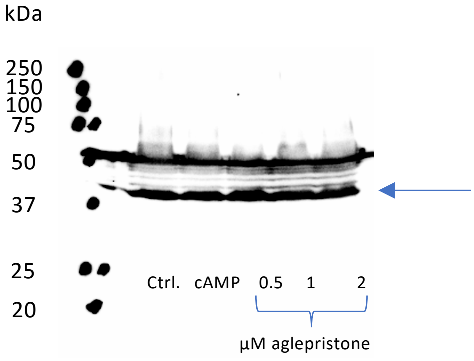

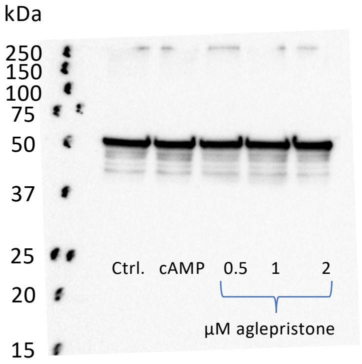

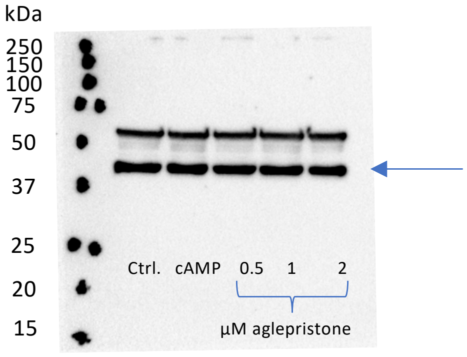

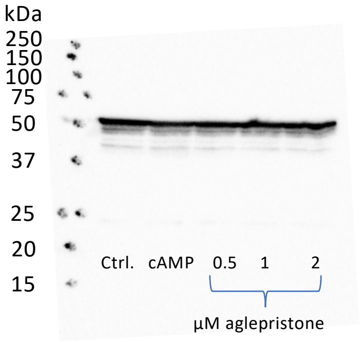

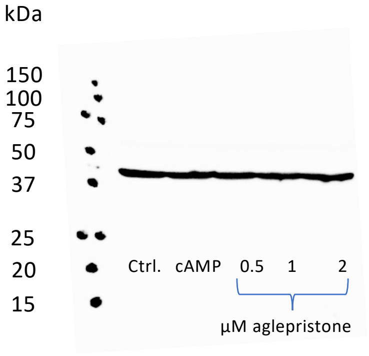

Mifepristone

α-SMA, 42kDa	ACTB, 45kDa

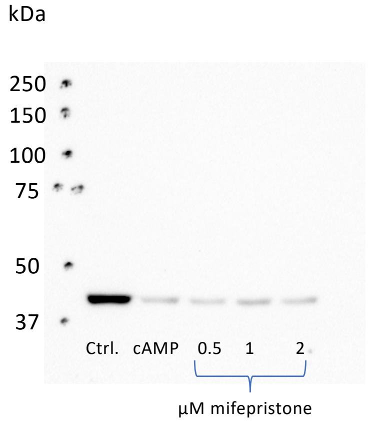

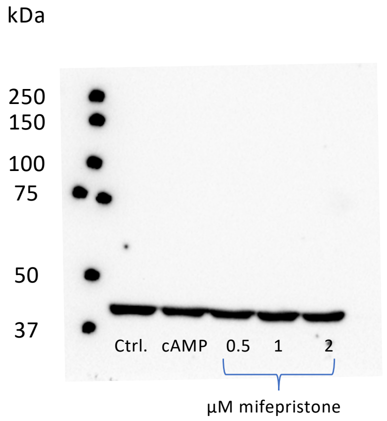

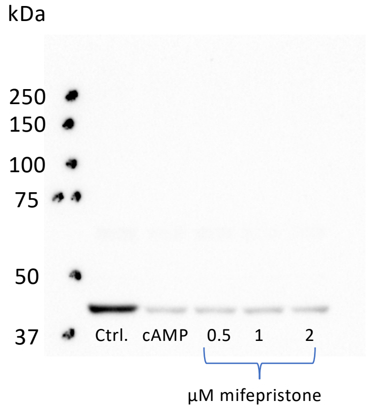

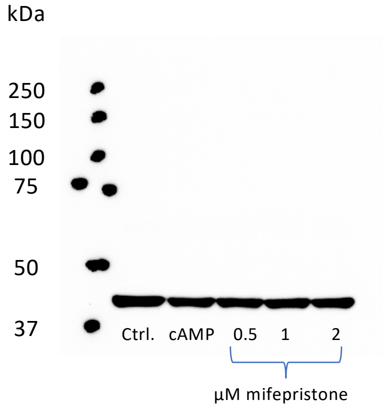

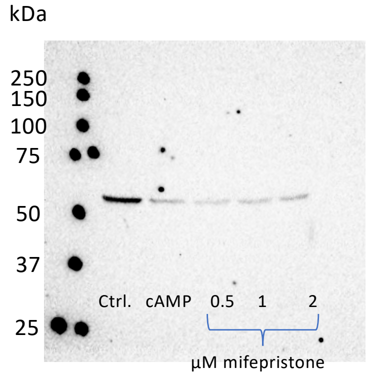

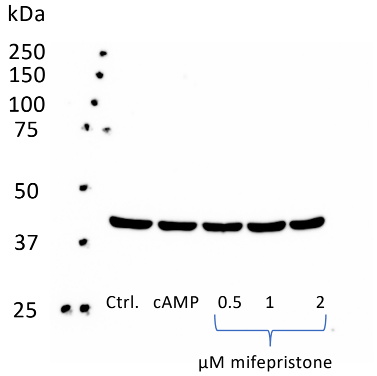

Aglepristone

α-SMA, 42kDa	ACTB, 45kDa

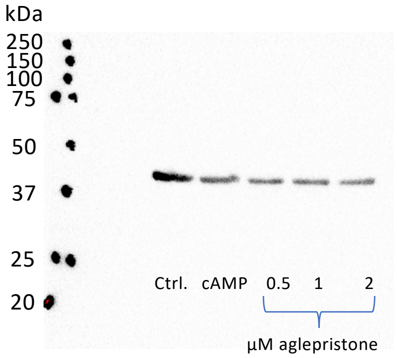

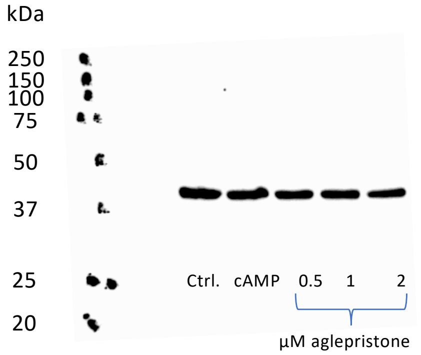

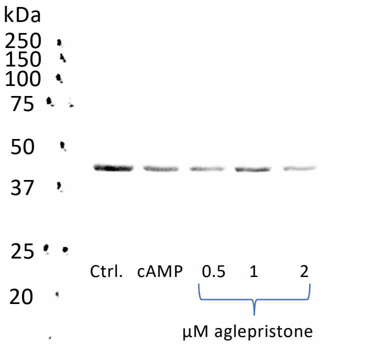

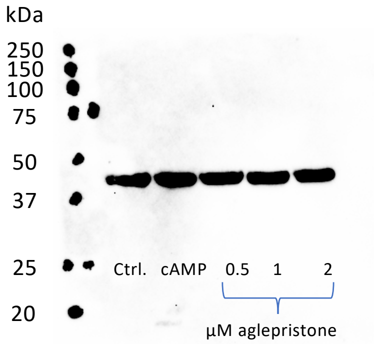

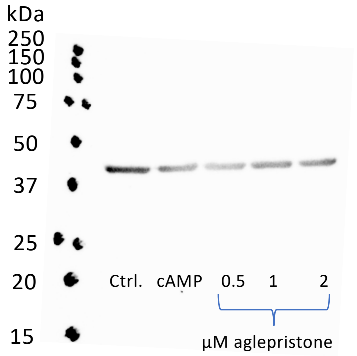

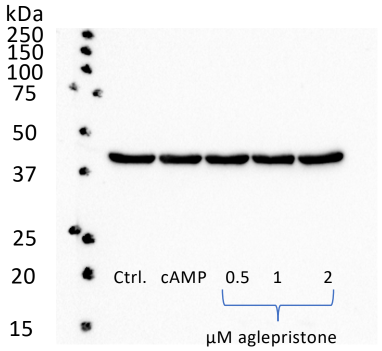

Mifepristone

PCNA, 29 kDa	ACTB, 45kDa

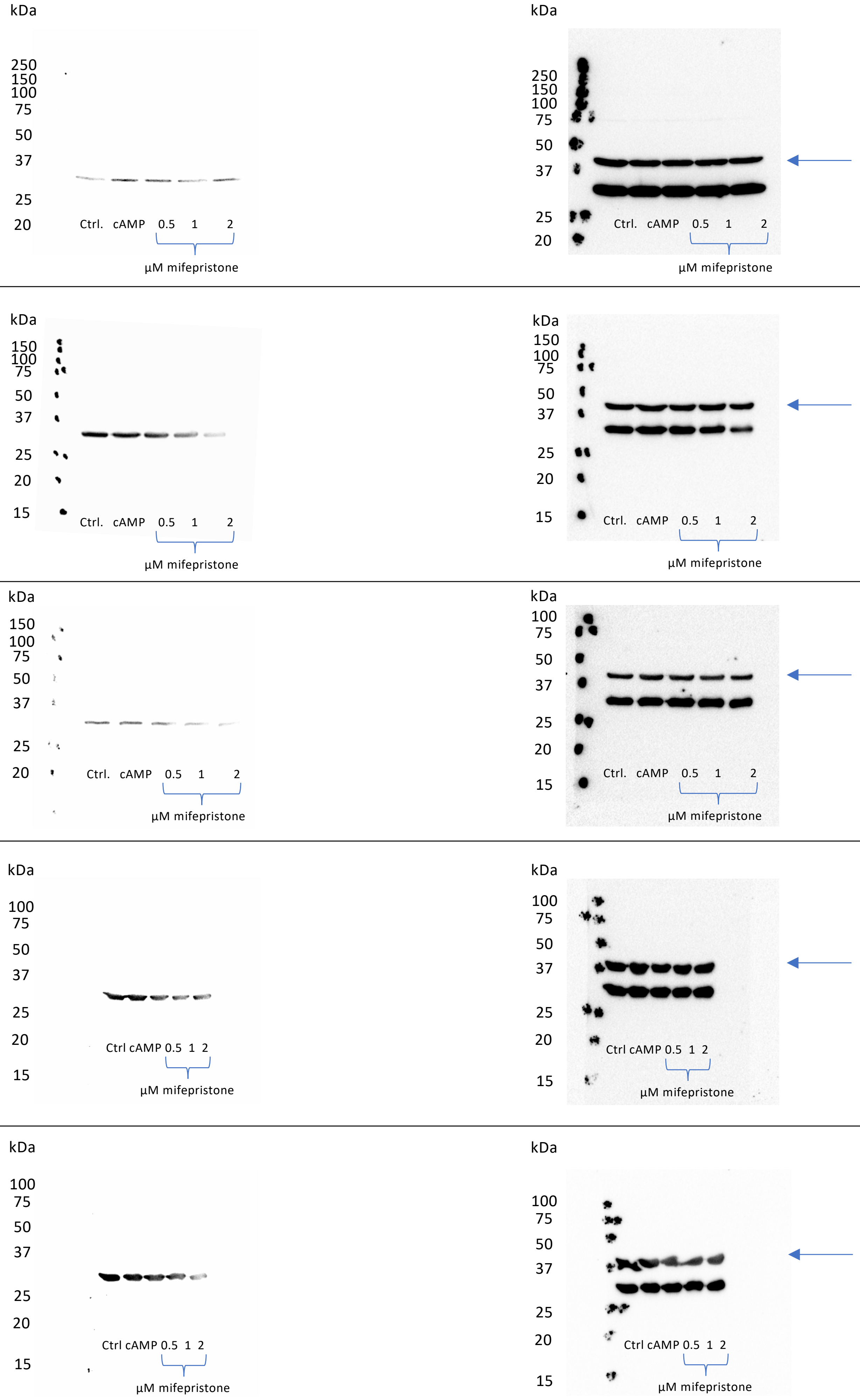

Aglepristone

PCNA, 29kDa	ACTB, 45kDa

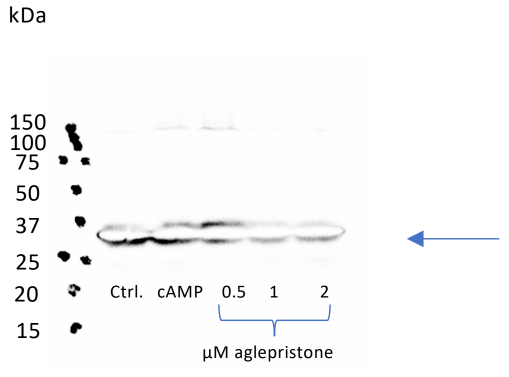

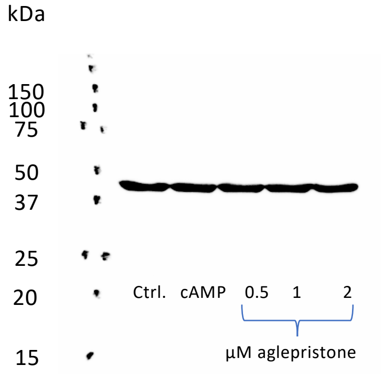

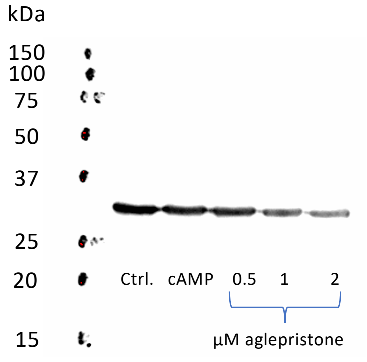

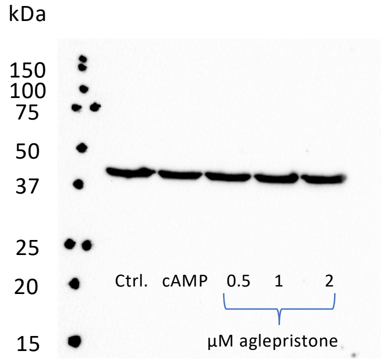

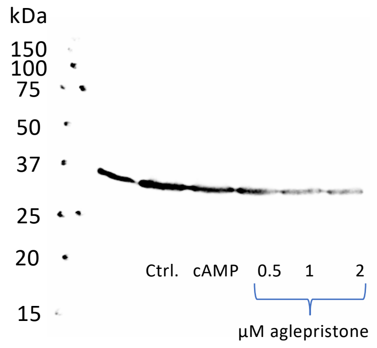

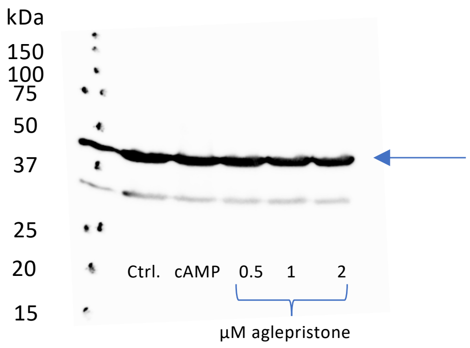

Mifepristone

a-CASP3, 20kDa	ACTB, 45kDa

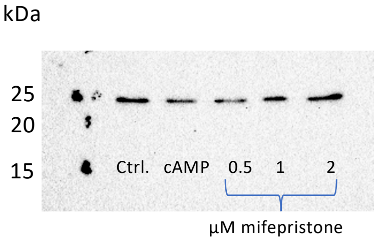

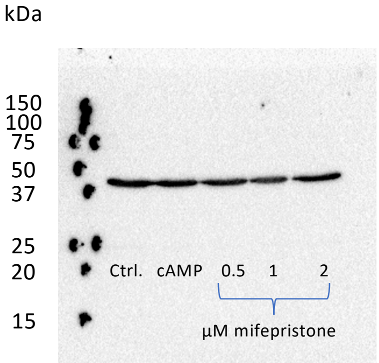

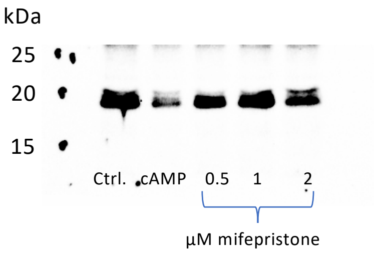

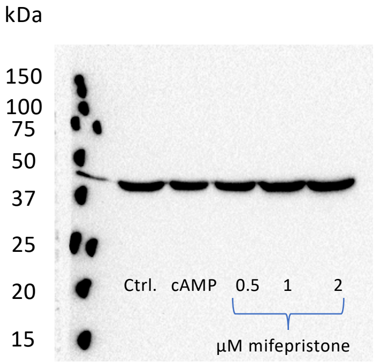

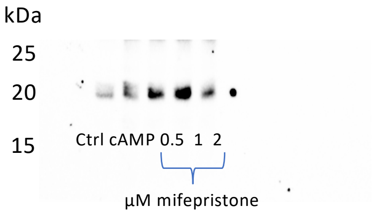

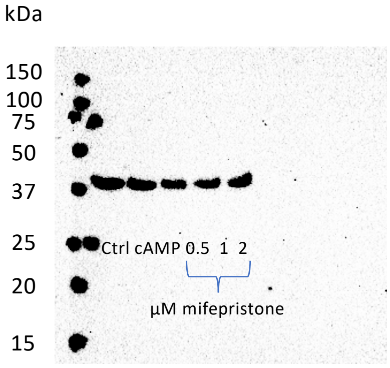

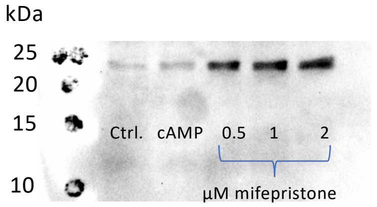

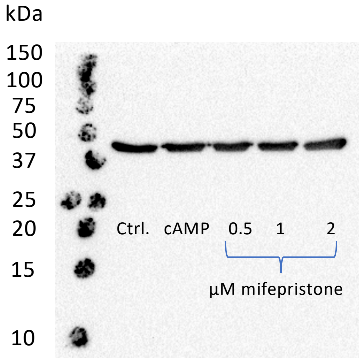

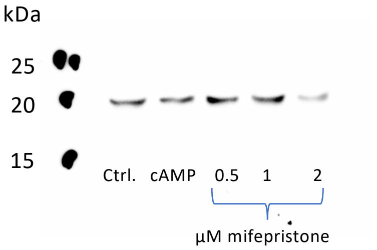

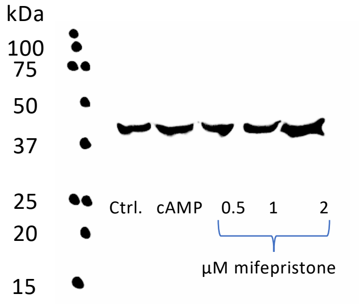

Aglepristone

a-CASP3, 20kDa	ACTB, 45kDa

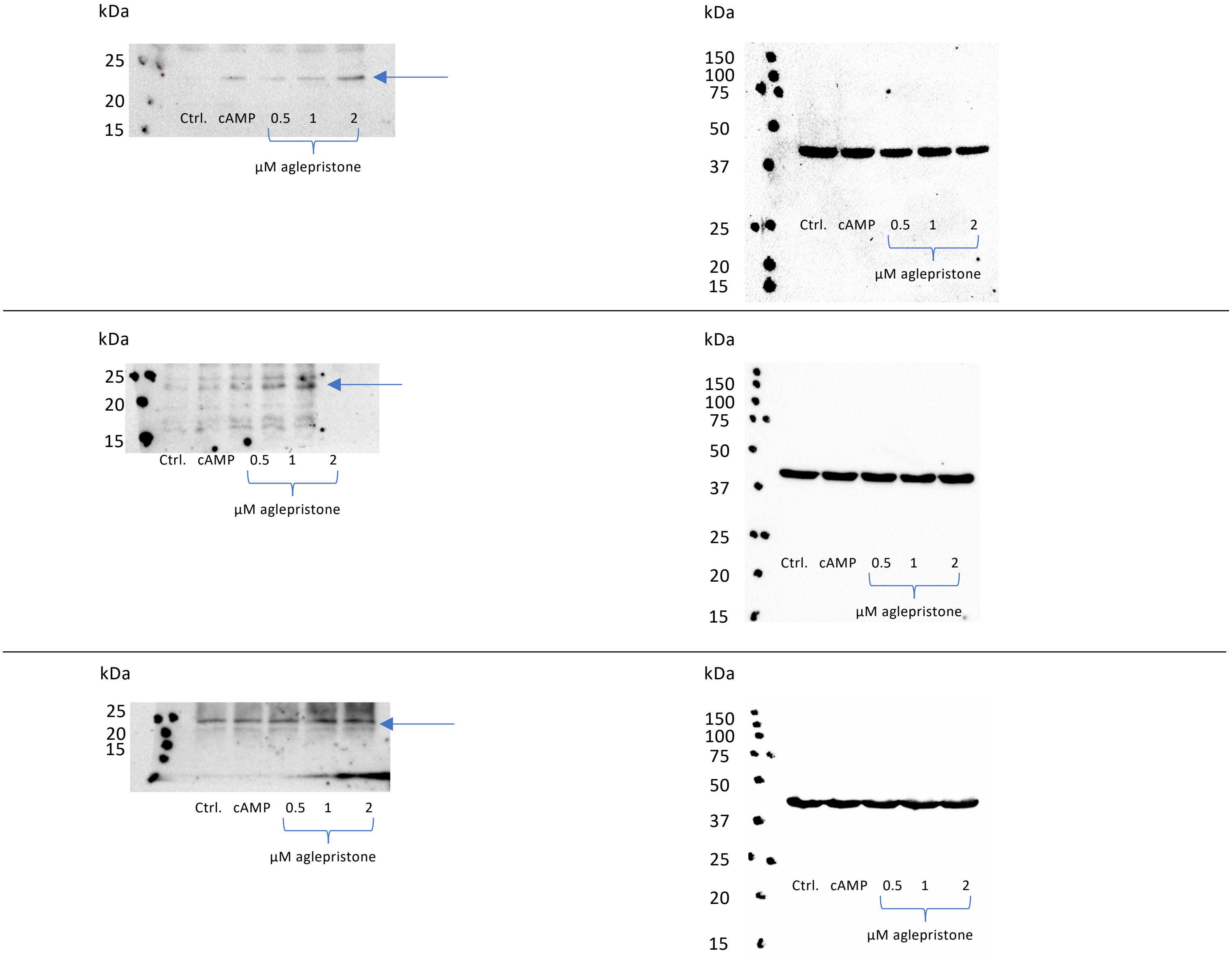

Supplement: Supplementary file 1 [file animals-12-00798-s001.zip › animals-1580737-supplementary.pdf]
